# The Halogenation Effects of Electron Acceptor ITIC for Organic Photovoltaic Nano-Heterojunctions

**DOI:** 10.3390/nano11123417

**Published:** 2021-12-16

**Authors:** Yu Wang, Cairong Zhang, Bing Yang, Lihua Yuan, Jijun Gong, Zijiang Liu, Youzhi Wu, Hongshan Chen

**Affiliations:** 1 Department of Applied Physics, Lanzhou University of Technology, Lanzhou 730050, China; yuwang199603@163.com (Y.W.); bingyang199702@163.com (B.Y.); yuanlh@lut.edu.cn (L.Y.); gongjijun@163.com (J.G.); 2 Department of Physics, Lanzhou City University, Lanzhou 730070, China; lzjcaep@126.com; 3 School of Materials Science and Engineering, Lanzhou University of Technology, Lanzhou 730050, China; youzhiwu@163.com; 4 College of Physics and Electronic Engineering, Northwest Normal University, Lanzhou 730070, China; chenhs@nwnu.edu.cn

**Keywords:** electronic structure, excitation, acceptor halogenation, organic photovoltaic, electron transfer

## Abstract

Molecular engineering plays a critical role in the development of electron donor and acceptor materials for improving power conversion efficiency (PCE) of organic photovoltaics (OPVs). The halogenated acceptor materials in OPVs have shown high PCE. Here, to investigate the halogenation mechanism and the effects on OPV performances, based on the density functional theory calculations with the optimally tuned screened range-separated hybrid functional and the consideration of solid polarization effects, we addressed the halogenation effects of acceptor ITIC, which were modeled by bis-substituted ITIC with halogen and coded as IT-2X (X = F, Cl, Br), and PBDB-T:ITIC, PBDB-T:IT-2X (X = F, Cl, Br) complexes on their geometries, electronic structures, excitations, electrostatic potentials, and the rate constants of charge transfer, exciton dissociation (ED), and charge recombination processes at the heterojunction interface. The results indicated that halogenation of ITIC slightly affects molecular geometric structures, energy levels, optical absorption spectra, exciton binding energies, and excitation properties. However, the halogenation of ITIC significantly enlarges the electrostatic potential difference between the electron acceptor and donor PBDB-T with the order from fluorination and chlorination to bromination. The halogenation also increases the transferred charges of CT states for the complexes. Meanwhile, the halogenation effects on CT energies and electron process rates depend on different haloid elements. No matter which kinds of haloid elements were introduced in the halogenation of acceptors, the ED is always efficient in these OPV devices. This work provides an understanding of the halogenation mechanism, and is also conducive to the designing of novel materials with the aid of the halogenation strategy.

## 1. Introduction

Organic photovoltaics (OPVs) have received extensive attention in recent years due to their light weight, low cost, semitransparency, flexibility, etc. The design and development of high-performance OPV devices are of great significance to solving the energy crisis [1,2,3]. Up to now, the power conversion efficiency (PCE) record of OPVs was over 18% [4,5,6,7]. Most of the electron processes for OPV power conversion happen in the active layer which is composed of both electron donor and acceptor materials. As the core of the OPV device, the changing active layer materials can tune photovoltaic performances [8,9]. In the past few years, the design and development of new electron donor and acceptor materials for OPV active layer have promoted the rapid improvement of PCE. Fullerene and its derivatives were usually used as electron acceptor materials due to their high electron affinity and electron mobility. However, their shortcomings of fullerenes limit their extensive application in OPV, including for complicated manufacturing processes, poor absorption in the visible and infrared region, and scare energy level adjustability. Non-fullerenes acceptors (NFAs) have many advantages over fullerenes, such as tunable energy levels and high molar absorption coefficients, resulting in a situation where NFAs have gradually replaced fullerenes as electron acceptors in OPV.

Backbone modification, side-chain engineering, and end-group substitution are common strategies for designing new electron donor and acceptor materials of OPV. Among these methods, halogen substitution has been shown to be effective for improving OPV performance. A fluorine atom is a type of halogen atom with the strongest electronegativity (3.98) and the smallest size (the van der Waals radius of 147 pm), which can effectively adjust the π-electron properties without causing unexpected steric hindrance. Experiments also proved that some chlorinated and brominated systems showed similar or stronger capability for adjusting π-conjugated electronic properties [10,11,12], which were caused by the larger dipole moments of C-Cl and C-Br bonds than that of a C-F bond [13]. Therefore, it is extremely important to research the influence of halogenation on materials’ physical properties and to explore the relationship between physical properties and photovoltaic performance.

Developing donor or acceptor materials by halogenation improved OPV performance [14,15]. Pei et al. found that the chlorinated backbone of a isoindigo-based polymer donor and acceptor (PC_71_BM) showed an appropriate degree of phase separation, reduced film crystallinity, and improved OPV performance (which was ascribed to the donor molecular stacking orientation [16]). Forrest et al. synthesized the BT-CIC by chlorinating BT-IC, and found that the chlorination caused the improved planarity of molecular geometry and significant red-shift of absorption spectrum (about 60 nm) with the 1.33 eV bandgap, resulting in an external quantum efficiency (EQE) of 75% in the wavelength region of 630–850 nm and of more than 11% of PCE [17]. Yan et al. reported that the C8-ITCC-Cl NFA which is chlorinated thienyls in C8-ITCC increased PCE from 10.8% to 12.7%, and found the chlorination of NFA induced the stronger noncovalent interactions which is favorable to improve the layered π-π stacking [18]. This highlighted the potential of halogenated thiophene end groups in NFA to improve PCE. Yasuda et al. designed a new type of NFA BDT-ID-X (X = F, Cl, Br, I), and found that subtle halogenations at the NFA end group can significantly affect the photoelectronic properties and film morphology [12]. Among them, Br and I substitutions can drastically reduce the surface free energies of donor/acceptor interfaces, forming fine bulk heterojunction (BHJ) for OPV. Hou et al. investigated the fluorinated electron donor PBDB-TF, fluorinated NFA BTP-4F, and chlorinated BTP-4Cl, and found that the halogenation can increase open-circuit voltage (V_OC_) and PCE (15.6% and 16.5%, respectively) by reducing energy losses [19]. The corresponding energy losses are 0.60 eV and 0.53 eV, and the non-radiative energy losses are 0.25 eV and 0.21 eV, respectively, which are lower than those of most reported OPVs. Cao et al. designed the NFA BDTBO-4F and BDTBO-4Cl through halogenation strategy and adopted PM6 as electron donor for fabricating OPV. Hole transfer at donor/acceptor interfaces was still efficient though the highest occupied molecular orbital (HOMO) offsets between the donor and acceptor are smaller than 0.1 eV, and the PM6:BDTBO-4F device exhibited a low charge recombination (CR) rate, high charge mobility, and suitable phase separation [20]. Also, halogenation at different substitution positions can cause changes in π-π stacking distance, supermolecular interaction, miscibility, and steric hindrance, which affect the aggregation behavior in blends [21,22,23,24,25,26,27].

In addition to experimental studies, many theoretical works were also conducted to understand the halogenation effects on OPV material properties [28,29]. Bredas et al. compared the electronic properties of polymers PBT4T-2OD and PffBT4T-2OD for understanding the correlation between solution temperature-dependent aggregation and solid-state stacking, and found that fluorination can enhance the planarity of the donor polymer backbone and increase the interaction among polymers, promoting molecule packing order for higher PCE [30]. Koehler et al. found that fluorination tends to reduce exciton binding energy (E_exb_), improve exciton dissociation (ED) efficiency, promote carrier mobility, restrict bimolecular CR, and reduce the resistance of bulk heterojunction [31]. Hou et al. reported that halogenation generates a larger difference of molecular surface average electrostatic potential (ESP) between the electron donor and acceptor [32]. Therefore, it induces a stronger intermolecular electric field at donor/acceptor interface which can increase ED efficiency and then enlarge the short-circuit current density (J_sc_) [33]. Based on the designed compounds, Mahmood et al. found that the fluorination of the end-groups increased the dipole moment difference between the ground state and the first excited state with the reduced E_exb_, and also enhanced electronic coupling for charge transport [34]. Our previous work indicated that electron donor halogenation can remarkably influence energy level alignment at heterojunction interfaces, and the improved PCE induced by donor halogenation can be mainly ascribed to the increased charge transfer (CT) excitation energies and suppression of CR processes [35].

The ITIC is a superstar of NFA and has been received extensive research attention since it inaugurated a new era of OPV [31,36,37]. The A-D-A type molecular architecture of ITIC, containing large planar conjugated backbone and strong electron-withdrawing end groups, promotes the π-electron delocalization and intramolecular charge transfer/transport. The study of the fluorinated and chlorinated ITIC by Hou et al. found that chlorination has a stronger ability to stabilize molecular energy levels and broaden the absorption spectrum than fluorination, and the position of halogen atoms can affect the crystallinity of halogenated ITIC [38,39]. Among the OPV devices based on bis-substituted halogenation (F, Cl, Br) of ITIC as NFAs and PBDB-T as electron donor, the fluorinated system generated the largest J_sc_, while the brominated system exhibited the highest FF (about 0.71) with a small amount of agglomeration area in the blended film, leading to the reduction of EQE and J_sc_ [40].

**Figure 1:**
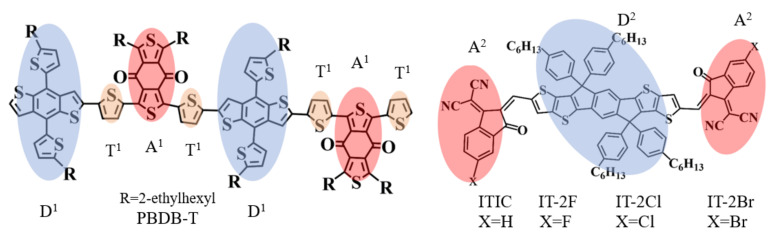
The molecular structures of electron donor PBDB-T, electron acceptors ITIC and IT-2X (X = F, Cl, Br). The polymer donor PBDB-T includes two repeat units. The molecules of electron donor and acceptor materials include electron donor fragments, acceptor fragments and thiophene units that connect the donor and acceptor fragments (labeled D, A, and T^1^ in the figure). The fragments D^1^ and D^2^ are electron donor moieties; the fragments A^1^ and A^2^ are electron acceptor moieties.

Overall, why halogenation of active layer materials usually improve OPV performance can be qualitatively attributed to two aspects. On the one hand, halogenation tunes electronic structures and excitation properties, including stabilizing molecular energy levels, enhancement of light absorption, increasing EQE and, thus J_sc_, and reducing energy losses and then increasing V_oc_ [41]. On the other hand, halogenation improves aggregation, packing order, crystallization and film morphologies due to the strengthened intramolecular or intermolecular interaction, achieving effective ED and charge transfer/transport [24,40,42,43,44]. However, the fundamental understanding of halogenation mechanisms, including the effects on electron processes at interface and OPV work principles, still remains uninvestigated. Here, to investigate acceptor halogenation effects, we selected PBDB-T:ITIC and PBDB-T:IT-2X (X = F, Cl, Br) as the model systems whose molecular structures are shown in Figure 1, and analyzed the molecular geometries, electronic structures, optical properties, excitation properties, ESP and the kinetics of electron processes at the heterojunction interfaces.

## 2. Computational Methods

The molecular structures of PBDB-T, ITIC and IT-2X (X = F, Cl, Br) were optimized at the level of ωB97XD/6-31G(d,p) [45,46]. Two repeat units were considered as the molecular structure of polymer donor PBDB-T in calculations, since the calculated frontier molecular orbitals (MOs) of polymer donor D18 whose molecular structure is similar to that of PBDB-T indicated that the frontier MOs are mainly localized on two repeat units [47]. Based upon the optimized molecular structures of PBDB-T, ITIC and IT-2X (X = F, Cl, Br), the PBDB-T:ITIC and PBDB-T:IT-2X (X = F, Cl, Br) heterojunction interfaces were modeled by using their complexes, in which the face-on configuration was considered due to the experimental results and the promoted π-π stacking [48]. To reduce the computational cost, the alkyl chains (-C_2_H_5_, -C_4_H_9_ in the polymer donor and -C_6_H_13_ in the acceptors) in the complexes were substituted with -CH_3_, since the side chain only affects the active layer morphology and does not affect other properties [49]; this simplification has been widely used in other works [31,50,51]. The constructed PBDB-T:ITIC and PBDB-T:IT-2X (X = F, Cl, Br) complexes were further optimized using ωB97XD/6-31G(d,p) methods. The excitation properties of donor and acceptor molecules, as well as the PBDB-T:ITIC and PBDB-T:IT-2X (X = F, Cl, Br) complexes, were calculated using time-dependent density functional theory (TDDFT) methods. The solid environment of OPV devices was simulated by self-consistent reaction field (SCRF) method with polarization continuum model (PCM) [52], requiring the static and dynamic dielectric constant (ε_s_ and ε_d_) to describe the polarization effects of solid environment. The ε_s_ and ε_d_ of models are 3.5 and 3.3, respectively.

TDDFT results heavily depend on the selected DFT functional. The long range corrected hybrid functional with the optimally tuned range separation parameter ω exhibited excellent performance in calculations of CT excitation. However, the optimally tuned range separation parameter in solid phase for long range corrected hybrid functional is quite small. Zheng et al. presented the optimally tuned screened range-separated hybrid (OT-SRSH) functional method, and gave the reasonable physical interpretation for optimally tuned range separation parameter [53]. In this work, the OT-SRSH functional method was applied with LC-PBE functional and 6-31G(d,p) basis sets for excitations, ESP and electronic coupling calculations. The following methods were adopted for optimally tuning ω [35,54,55]:

(1) $$E_{1}^{\omega }=|E_{D\left(HOMO\right)}^{\omega }+E_{D^{+}}^{\omega }-E_{D}^{\omega }|\;+\;|E_{A\left(HOMO\right)}^{\omega }+E_{A^{+}}^{\omega }-E_{A}^{\omega }|\;E_{2}^{\omega }=|E_{D\left(HOMO\right)}^{\omega }+E_{D^{+}}^{\omega }-E_{D}^{\omega }|\;+\;|E_{A^{-}\left(HOMO\right)}^{\omega }+E_{A}^{\omega }-E_{A^{-}}^{\omega }|\;E^{\omega }=E_{1}^{\omega }+E_{2}^{\omega }$$

where $E_{D\left(HOMO\right)}^{\omega }$, $E_{A\left(HOMO\right)}^{\omega }$, $E_{A^{-}\left(HOMO\right)}^{\omega }$ are the HOMO level to the neutral donor, acceptor at neutral state and acceptor at anion state, respectively. The $E_{D^{+}}^{\omega }$, $E_{A^{+}}^{\omega }$, $E_{A^{-}}^{\omega }$ are the single-point energy of the donor at cation state, acceptor at cation state, and acceptor at anion state, respectively. The $E_{D}^{\omega }$ and $E_{A}^{\omega }$ are the neutral state energy of the donor and acceptor, respectively. The optimally tuned ω is 0.155, 0.155, 0.167, 0.144, 0.155 *Bohr*^−1^ for PBDB-T, ITIC and IT-2X (X = F, Cl, Br), respectively.

In order to investigate the effect of acceptor halogenation on the electronic processes, the rate of CT, CR, and ED were calculated using Marcus’ theory. The rate constant K can be calculated as following formula,

(2) $$K=\sqrt{\frac{4\pi^{3}}{h^{2}\lambda k_{B}T}}|V{|}^{2}exp\left[-\frac{{\left(\Delta G+\lambda \right)}^{2}}{4\lambda k_{B}T}\right]$$

where λ is the reorganization energy, including the internal-reorganization energy λ_i_ and the external-reorganization energy λ_ext_, *V* represents the electronic coupling between the initial state and the final state, ΔG is the free energy change, $K_{B}$ is Boltzmann’s constant, h is Planck’s constant, and T is temperature (300 K was adopted). The λ_i_ were estimated from the energies of donor and acceptor molecules [55,56]. The two-sphere model was adopted to calculate the λ_ext_ [57]. The electronic couplings of CR processes were calculated using the Generalized Mulliken Hush (GMH) model [58,59], and the electronic couplings of CT and ED processes were calculated using two-state model [60].

The DFT and TDDFT calculations in this work were conducted using Gaussian 09 software [61]. The Multiwfn software was applied for the quantitative analysis of ESP, charge density difference (CDD), the transferred charges (Δq) and CT distance (Δd) [62,63,64].

## 3. Results and Discussion

### 3.1. PBDB-T, ITIC and IT-2X (X = F, Cl, Br) Properties

The unit of PBDB-T with D-π-A type structure contains electron donor moiety based on benzo[1,2-b:4,5-b′]dithiophene (BDT) core, thienyl as π-spacer, and acceptor fragment based upon 5,7-bis(2-ethylhexyl)benzo[1,2-c:4,5-c′]dithiophene-4,8-dione (BDD) moiety, labeled as D^1^, T^1^, and A^1^ in Figure 1, respectively. For the A-D-A structure ITIC and IT-2X (X = F, Cl, Br), the acceptor fragments are 3,9-bis(2-methylene-(3-(1,1-dicyanomethylene)-indanone)) (DCI), and the 5,5,11,11-tetrakis(4hexylphenyl)-dithieno[2,3-d:2′,3′-d′]-s-indaceno[1,2-b:5,6-b′]-dithiophene (DID) fragment plays the role of donor. The optimized molecular structures of PBDB-T, ITIC and IT-2X (X = F, Cl, Br) are shown in Figure S1 in Supporting Material (SM), where the atomic serial numbers are also labeled. Table 1 lists the bond lengths, bond angles, and dihedral angles of the optimized PBDB-T, ITIC and IT-2X (X = F, Cl, Br) molecules, which can describe the distances and orientations among different moieties. More detailed geometric data are listed in Table S1 in SM. The dihedral angles between T^1^ and BDD/BDT moieties in PBDB-T indicate the non-planar main chain and significant torsion among these fragments, which result from the steric hindrance of alkyl in side chain and dione in BDD moiety. For ITIC and IT-2X (X = F, Cl, Br), the DCI and DID are planar fragments, cyano groups are coplanar with DCI, and small torsion angles between DCI and DID (3.8°, −7.6°, −7.8°, 3.2° in ITIC and IT-2X (X = F, Cl, Br), respectively) indicate their quasi-planar structures, meaning that introducing halogen atom into DCI fragments at end sites cannot significantly affect the planarity of backbone. The molecular backbone planarity can promote electron delocalization and enhance intramolecular CT. Moreover, the intramolecular hydrogen bonds between the O in DCI and the adjacent H in DID enhance the backbone planarity of ITIC and IT-2X (X = F, Cl, Br). The average C-X (X = H, F, Cl, Br) bond lengths are 1.09, 1.34, 1.74, and 1.89 Å, respectively. The increase of C-X bond lengths is caused by the size of the halogen atoms. Hence, considering the end sites halogenation and the variations of torsion angles between DCI and DID fragments, the halogenation of ITIC cannot significantly reduce backbone planarity and increase steric hindrance. However, due to the strong electronegativity of halogen atom, halogenation can induce stronger inter-molecular hydrogen bonds, which are favorable for molecular packing order, and then for efficient charge transport/transfer processes.

Table 2 shows the energies of HOMO, the lowest unoccupied molecular orbital (LUMO) and other MOs, as well as the HOMO-LUMO gap (H-L_gap_) of PBDB-T, ITIC, and IT-2X (X = F, Cl, Br) molecules. The data indicate that the fluorination, chlorination, and bromination downshift the HOMO about 0.03, 0.04, and 0.05 eV, respectively, and reduce LUMO about 0.02, 0.08, and 0.08 eV, respectively, corresponding to slight variation of H-L_gap_, agreeing experimental tendency of HOMO and LUMO variations by halogenation that were estimated by using electrochemical cyclic voltammetry measurements [40]. Also, halogenation of ITIC cannot effectively modify the energy differences between HOMO and HOMO-1, as well as between LUMO and LUMO+1, which were introduced as descriptors in machine-learning study of OPV materials [65]. Compared with ITIC, the downshift of HOMO and LUMO energies of IT-2X (X = F, Cl, Br) can be understood from the electronegativity of halogen atoms [66,67]. The strong electronegativity of halogen atoms causes more positive charges populated on the phenyl ring that bonded with halogen atoms, resulting in more electron localization and polarization effects [31]. Meanwhile, strong electronegativity of the halogen atom also changes the orbital hybridization of carbon atoms that bind with halogen atoms. The LUMO energy offset between donor and acceptor is usually considered as driving force for ED, while the energy offset between the LUMO of acceptor and the HOMO of donor is regarded as the driving force for CR. In terms of the data listed in Table 2, the halogenation of ITIC increase ED driving force and reduce CR driving force.

**Figure 2:**
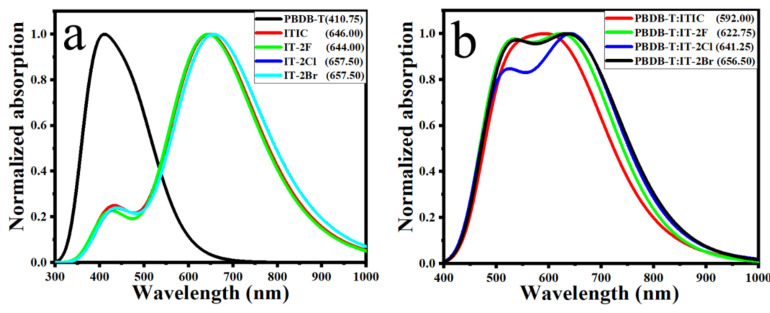
The simulated absorption spectra of electron donor PBDB-T, electron acceptors ITIC and IT-2X (X = F, Cl, Br) (**a**) and the donor/acceptor complexes (**b**) in solid phase. The half-width at half-maximum of 0.330 eV (**a**) and 0.255 eV (**b**) were applied for absorption spectra simulations. The wavelengths (in nm) at absorption maxima are marked in the figure. (LC-PBE/6–31G**; *α* = 0.200, *β* = 0.086; *ε_s_* = 3.5, *ε_d_* = 3.3).

It was observed that the electron donor PBDB-T film exhibited good complementary absorption with those of acceptor ITIC and IT-2X (X = F, Cl, Br) [40]. PBDB-T has a good absorption characteristic in the region of 450–650 nm with absorption peaks at 615.5 nm and 567.5 nm, respectively. The absorption ranges of ITIC and IT-2X (X = F, Cl, Br) are between 600–815 nm. The complementary absorption of donor and acceptor in organic heterojunction are favorable to improve J_sc_ by enhancing photon harvest [68]. The simulated absorption spectrum presented in Figure 2 well reproduced experimental results, including absorption profile, the wavelength at absorption maxima (λ_max_) and relative shift among IT-2X (X = F, Cl, Br). For instance, the λ_max_ of IT-2F is blue shifted 13.5 nm relative to those of IT-2Cl and IT-2Br, agreeing well with that of experimental 14 nm excellently [40].

**Figure 3:**
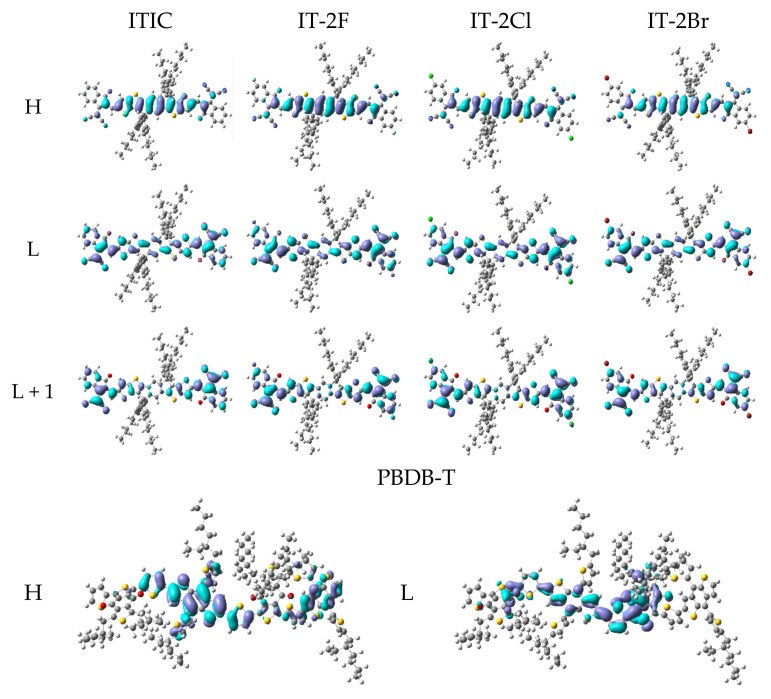
The selected frontier molecular orbitals for the ITIC, IT-2X (X = F, Cl, Br) and PBDB-T. (LC-PBE/6-31G**, ω = 0.155, 0.167, 0.144, 0.155, 0.155 *Bohr*^−1^; *α* = 0.200, *β* = 0.086; *ε_s_* = 3.5, *ε_d_* = 3.3; H = HOMO, L = LUMO).

Table 3 lists the main transition configurations, excited states characters (ESC), oscillator strengths (*f*), excitation energies and wavelengths of the selected excited states. More detailed data of PBDB-T, ITIC and IT-2X (X = F, Cl, Br) are given in Table S2 in SM. The related MOs are presented in Figure 3, and more MOs are shown in Figure S2 in SM. The HOMO of PBDB-T is mainly contributed by BDT and thienyl fragments, whereas the LUMO is mainly composed of BDD, thienyl and BDT fragments. Hence, in terms of transition configurations and MOs, the main character of PBDB-T’s S1 state is hybridized of CT and LE that is localized in donor fragment BDT (DLE). The PBDB-T’s other excited states that are listed in Table 3 are CT or hybridizations of CT and DLE. Considering the *f* of PBDB-T’s S1 state, the BDT and thienyl fragments are effective moieties in determining its light harvest efficiency. For ITIC and IT-2X (X = F, Cl, Br), their HOMOs are mainly contributed by DID fragment, while their LUMOs extend to DCI fragments. According to S1 transition configurations of ITIC and IT-2X (X = F, Cl, Br), the overlap between HOMO and LUMO means the LE of DID, which belongs to DLE. However, the relocation between HOMO and LUMO suggests CT excitations. Hence, the S1 states of ITIC and IT-2X (X = F, Cl, Br) hybridize the CT and DLE. The other excited states listed in Table 3 are also hybrid excitations. Since LE usually corresponds to large *f* that determine light harvest efficiency, DID is an effective chromophore for light harvest of ITIC and IT-2X (X = F, Cl, Br). CT excitations are also expected for increasing current density, efficient ED, and charge transfer/transport. The hybrid excitations combine the merits of CT and LE. Furthermore, halogenation doesn’t significantly influence on excitation properties (transition configurations and ESC, etc.) of IT-2X (X = F, Cl, Br) except the slight change of excitation energies. The S1 excitation energies indicate that fluorination slightly increases excitation energy, while the chlorination and bromination slight reduce excitation energies. This can be understood from the transition configurations and halogenation effects on frontier MO energies. Moreover, there are some quasi-degenerated excited states (see Table S2 in SM), for instance, S11 and S12, S13, and S14 of IT-2X (X = F, Cl, Br). The CT of these excited states can be enhanced due to coherence among quasi-degenerated states.

The CDD, CT distances Δd and the transferred charges Δq for the selected low-lying excited states of ITIC and IT-2X (X = F, Cl, Br) are shown in Figure S3 in SM. The interpenetrating of electron density increment/decrement regions and small Δq support these excited states are hybrid excitations of CT and LE. Moreover, the CDD of ITIC and IT-2X (X = F, Cl, Br) suggest that the DCI and DID fragments are electron acceptor and donors, respectively, and the phenyl moieties in side chains also play the role of electron donor. In addition, the A-D-A type structure and symmetry of ITIC and IT-2X (X = F, Cl, Br) result into small Δd of the excited states presented in Figure S3.

**Figure 4:**
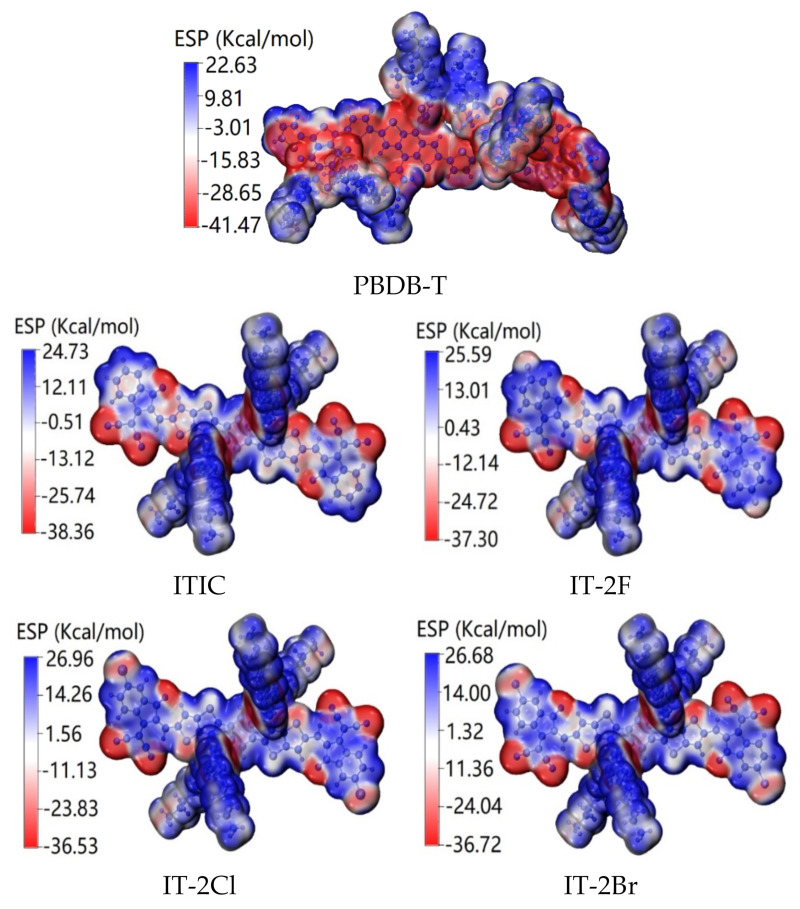
Electrostatic potentials (ESP) distributions of ITIC, IT-2X (X = F, Cl, Br) and PBDB-T molecules. The red (blue) color represents the low (high) potential areas. (LC-PBE/6-31G(d,p); *α* = 0.200, *β* = 0.086; *ε_s_* = 3.5, *ε_d_* = 3.3; ω = 0.155, 0.155, 0.167, 0.144, 0.155 *Bohr*^−1^).

Figure 4 shows the ESP of ITIC, IT-2X (X = F, Cl, Br), and PBDB-T molecules. For PBDB-T, the low ESP is mainly contributed by backbone, and the high ESP is dominated by side chains. For ITIC and IT-2X (X = F, Cl, Br), the high ESP are mainly contributed by alkyl in side-chains, and the low ESP mainly concentrate on O, S, and N atoms in backbone. Moreover, halogenation reduces the ESP at substitution sites. The average ESP values are −48.6, 63.8, 85.3, 105.9 and 106.4 Kcal/mol for PBDB-T, ITIC, IT-2F, IT-2Cl, and IT-2Br, respectively. Since the increased average ESP value of electron acceptor can increase the electron accepting ability [69], the halogenation of ITIC promotes their electron accepting ability, which is favorable for enhancing CT between donor-acceptor complexes [32]. Furthermore, halogenation of ITIC also generate the enlarged ESP differences between donor and acceptors, and further induce stronger interface electric field that can promote the ED and CT at the donor-acceptor interface [33]. Moreover, the enlarged ESP difference between donor and acceptors may also suppressing non-radiation recombination (which was regarded as one of the important energetic losses route in OPV) [70].

### 3.2. PBDB-T:ITIC and PBDB-T:IT-2X (X = F, Cl, Br) Properties

**Figure 5:**
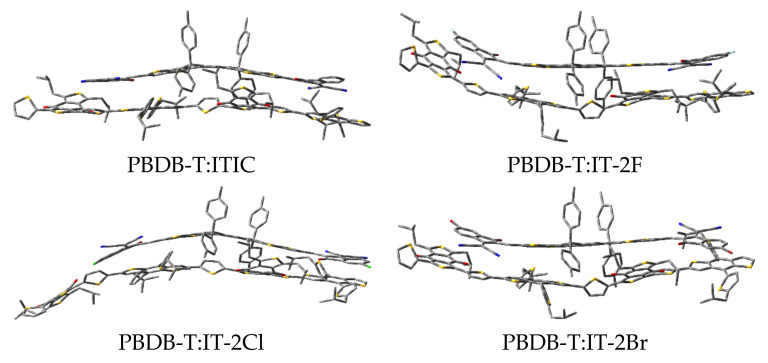
The optimized structures of PBDB-T:ITIC and PBDB-T:IT-2X (X = F, Cl, Br) complexes. (ωB97XD/6-31G**).

The optimized structures of PBDB-T:ITIC and PBDB-T:IT-2X (X = F, Cl, Br) complexes are shown in Figure 5, and the selected geometric parameters are given in Table 4. Compared with those of the corresponding geometric parameters of the ITIC and IT-2X (X = F, Cl, Br) molecules, the changes in bond lengths and angles are smaller than 0.001 Å and 1° for the corresponding electron acceptors in complexes, respectively. The data in Table 4 indicate that the non-bond intermolecular interaction reduce the backbone planarity of ITIC and IT-2X (X = F, Cl, Br) in complexes. The distance between the mass centers of donor and acceptor in PBDB-T:ITIC and PBDB-T:IT-2X (X = F, Cl, Br) complexes are 4.32, 3.03, 5.86, and 3.19 Å, respectively. The maximum dihedral angle variations in complexes locate at the torsion between the donor DID and acceptor DCI fragments in ITIC and IT-2X (X = F, Cl, Br), and the corresponding values are 11°, 19°, 9°, 13°, respectively. Similarly, relative to those of isolated PBDB-T molecules, the changes of bond lengths and angles in complexes are very tiny. Due to the absence of intramolecular hydrogen bonds in PBDB-T, the PBDB-T’s backbone is more flexible than ITIC and IT-2X (X = F, Cl, Br), corresponding to several remarkable dihedral angle changes of PBDB-T in complexes. The intermolecular non-bonding interactions can be evaluated from the binding energies (E_b_) of the complexes. The calculated results show that halogenation increases E_b_ (see Table 5), indicating that halogenation enhances the non-bonding intermolecular interaction between electron donor and acceptor, which is beneficial to improve the morphology regioregularity that was approved by relatively smooth surfaces of PBDB-T:IT-2X (X = F, Br) blended films [40]. The data of quadrupole moments of ITIC and halogenated systems (see Table S5 in SM) indicate halogenation results into an increase of the quadrupole, and then contribute to enhance quadrupole interaction between the donor and acceptor molecules in complexes [71]. Moreover, the large E_b_ may enhance interface stability and improve resistance to thermal/light stress [72].

Table 5 also gives the HOMO and LUMO energies, as well as the H-L_gap_ values of the PBDB-T:ITIC and PBDB-T:IT-2X (X = F, Cl, Br) complexes. Similar to that of ITIC and IT-2X (X = F, Cl, Br) molecules, the fluorination slightly elevates the LUMO of complex with broadening H-L_gap_, whereas the chlorination and bromination induce down-shift of LUMO of complexes, corresponding to H-L_gap_ reductions. Among these complexes, the H-L_gap_ of PBDB-T:IT-2Cl complex is the smallest. According to Shockley-Queisser theory, the lowering H-L_gap_ tends to reduce energy loss [73]. Hence, chlorination and bromination may be helpful to suppress energy loss.

The absorption spectra of complexes that were presented in Figure 2b indicate that the halogenation not only induce the red-shift about 30–65 nm, but also enhance absorption in longer wavelength regions. The enhanced optical absorption is expected for prominent OPV materials since it can improve the utilization of photon energy from solar radiation.

Organic materials with a smaller exciton binding energy (E_exb_) usually show better charge separation efficiency, which impacts the free charge carrier generation in photon-to-electricity conversion of OPV. The calculated E_exb_ of PBDB-T:ITIC and PBDB-T:IT-2X (X = F, Cl, Br) complexes are 0.63, 0.59, 0.62, and 0.60 eV, respectively, meaning that halogenation reduces E_exb_. The small E_exb_ is favorable to reduce interfacial energy offsets toward efficient ED for free charge carrier generation in OPV [74,75,76]. In terms of the related parameters for calculating E_exb_ listed in Table S6, the main reason for E_exb_ reduction is halogenation increases electron affinity.

**Figure 6:**
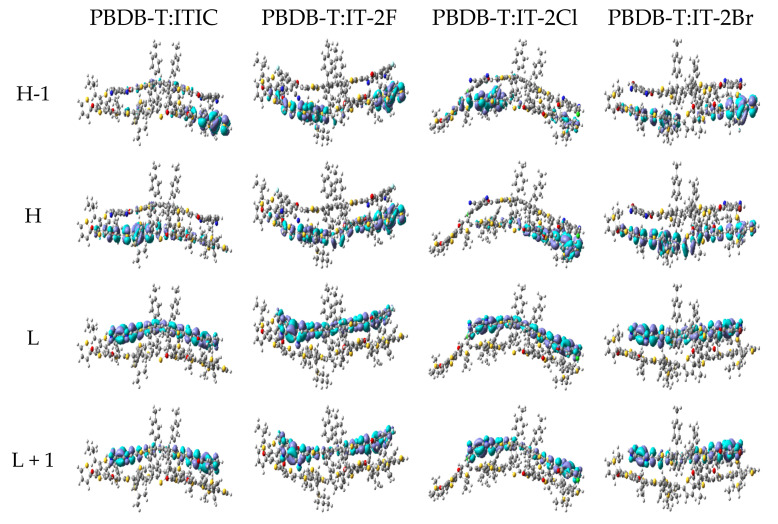
The selected frontier molecular orbitals for PBDB-T:ITIC and PBDB-T:IT-2X (X = F, Cl, Br) complexes. (LC-PBE/6-31G**, ω = 0.155, 0.167, 0.144, 0.155 *Bohr*^−1^; *α* = 0.200, *β* = 0.086; *ε_s_* = 3.5, *ε_d_* = 3.3; H = HOMO, L = LUMO).

Table 6 lists the excitation energies, corresponding oscillator strengths, excitation wavelengths, excited state characteristics and the main transition configurations of PBDB-T:ITIC and PBDB-T:IT-2X (X = F, Cl, Br) complexes. Details of more excited states are listed in Table S4 in SM. Figure 6 presents the selected MOs of PBDB-T:ITIC and PBDB-T:IT-2X (X = F, Cl, Br) complexes. More MOs are showing in Figure S4 in SM. According to transition configurations and MOs, the excited states’ characters were assigned. The S1 states of all the complexes in this work are CT excitations, and the energies of local excitation in acceptor (ALE) and in donor (DLE) are higher than that of CT states, indicating energetic permission for ED. Comparing the CT state energy of PBDB-T:ITIC complex, the fluorination and bromination increase CT energy 0.11 and 0.02 eV, respectively, while the chlorination reduces CT energy 0.13 eV. Combined with the empirical formula of V_OC_ [77], the large CT excitation energy is beneficial to improve the V_OC_. Meanwhile, the halogenation effects on CT excitation energies of these complexes are similar to that on H-L_gap_, which can be understood from transition configurations and weak intermolecular interaction between PBDB-T and acceptor in complexes. In addition, couple of excited states with higher energy of these complexes exhibit hybridization of CT and local excitation (see Table S4 in SM), which are favorable to charge carrier generation by hot ED processes [78].

**Figure 7:**
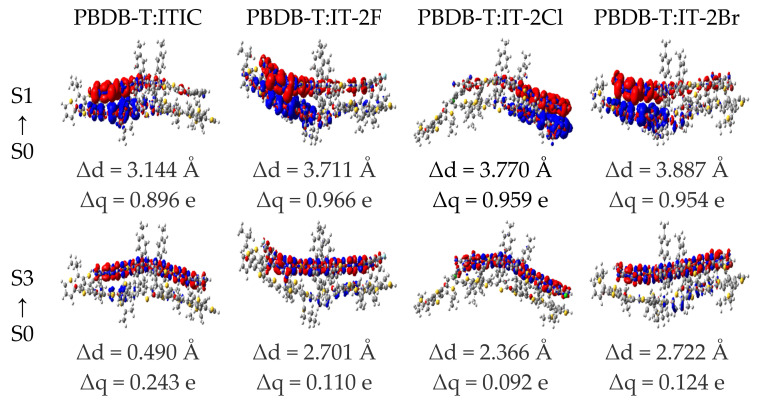
Charge density difference for the low-lying excited states of PBDB-T:ITIC and PBDB-T:IT-2X (X = F, Cl, Br) complexes. The regions colored in red (blue) indicate the increase (decrease) of electron density during excitation processes, respectively. The charge transfer distance Δd and the transferred charges Δq are shown. (LC-PBE/6-31G**, ω = 0.155, 0.167, 0.144, 0.155 *Bohr*.

The Δq and Δd of the PBDB-T:ITIC and PBDB-T:IT-2X (X = F, Cl, Br) complexes are also calculated to quantitatively describe the CT and LE of the complexes. The CDD, Δq and Δd of the selected excited states are given in Figure 7 (more details to see Figure S5 in SM). Apparently, the Δq labeled in Figure 7 and Figure S4 support the assignments of excited state characters. The halogenation of the complexes increases Δq of CT excitations. The Δd for most of excited CT states are in range of typical intermolecular distances between donor and acceptor in organic photovoltaic heterojunctions. Furthermore, the order of Δq related to S1 states of the halogenated complexes is same as that of experimental J_SC_ [40]. The Δq was demonstrated as one of the critical factors to determine J_SC_ [79].

### 3.3. Rate Constants of Charge Transfer, Exciton Dissociation and Charge Recombination

Table 7 lists the calculated λ (including λ_i_ and λ_ext_), ∆G and *V* of the CT, CR and ED processes, which are required for calculating rate constants. Tables S6–S7 in SM provide the detailed parameters for calculating *V*. Since the halogenation site is at the end group of ITIC, the halogenation influences on λ are very tiny for each process (<0.01 eV). Meanwhile, the halogenation effects on ∆G are smaller than 0.1 eV for each process. As to *V*, apart from chlorination of ED process, the halogenation reduce *V* for each process. Compared with the rates of PBDB-T:ITIC complex, the fluorination increase CT rate and decrease CR rate. However, both the chlorination and bromination reduce CT rates and increase ED rates. For ED processes, the fluorination and chlorination decrease ED rates, while the bromination increases ED rate. In real OPV devices, CT, CR, and ED processes are competitive, resulting into ultimate performance. Considering the CT and ED rates are more than 13 orders larger than CR rates, meaning the efficient ED in these OPV devices. In the experiment, the ratio of the photocurrent density (J_ph_) and its saturation (J_sat_) is used to evaluate exciton dissociation efficiency and charge collection [40]. Charge collection is mainly determined by cathode/anode layers and their interfaces. Due to the same OPV architecture and experimental condition, the J_sat_ must be same. Hence, the J_ph_/J_sat_ ratios expose the ED efficiency. The J_ph_/J_sat_ ratios of PBDB-T:ITIC, PBDB-T:IT-2F, PBDB-T:IT-2Cl and PBDB-T:IT-2Br were 93.3%, 92.2%, 90.6%, and 94.8%, respectively [40], agreeing well with the tendency of the calculated ED rates.

## 4. Conclusions

In this work, based on the quantum chemical calculations and the selected model systems which include donor PBDB-T, acceptors ITIC and IT-2X (X = F, Cl, Br), the halogenation mechanism and its effects on photovoltaic performances were studied. The main conclusions are as followings:

Halogenation of electron acceptor ITIC in OPV heterojunction slightly affect the molecular geometric structures, MO energy levels, optical absorption spectra, exciton binding energies, and excitation properties. However, the halogenation of ITIC significantly enlarge the ESP difference between electron acceptor and donor PBDB-T with the order from fluorination and chlorination to bromination that can induce an electric field at the heterojunction interface, and the halogenation can also increase the transferred charges of CT states for the complexes, corresponding to the increase of J_sc_. Meanwhile, the halogenation effects on CT energies and electron processes rates depend on different elements. No matter which kinds of elements were introduced in the halogenation of the acceptor, the ED is always efficient in these OPV devices since the ED rates are more than 13-fold larger than CR rates. Hence, the main halogenation effects on OPV performances can be ascribed to its influences on ESP, transferred charges, and electron processes rates.

This work provides help in understanding the mechanism of halogenation influences on OPV performances of acceptor material properties, and is also conducive to the design of new high-performance acceptor materials with the aid of halogenation strategy.

## Figures and Tables

**Table 1:** The selected geometrical parameters, including bond length (in Å), bond angles (in °) and dihedral angles (in °) which were defined by two, three, and four atoms. (ωB97XD/6-31G**).

Definition	Bond Length	Definition	Bond Angles	Definition	Dihedral Angles
ITIC					
1–2	1.085	1-2-3	120.1	6-15-17-18	3.8
9–10	1.370	11-10-12	112.2	8-9-10-11	1.8
66–67	1.370	74-73-65	120.1	64-66-67-69	−177.5
73–74	1.085	69-67-68	112.2	61-60-58-57	3.8
IT-2F					
1–2	1.335	1-2-3	119.2	6-15-17-18	−7.6
9–10	1.370	11-10-12	112.6	8-9-10-11	−2.6
66–67	1.370	74-73-65	119.2	64-66-67-69	176.1
73–74	1.335	69-67-68	112.6	61-60-58-57	−7.6
IT-2Cl					
1–2	1.741	1-2-3	119.5	6-15-17-18	−7.8
9–10	1.370	11-10-12	112.6	8-9-10-11	−2.8
66–67	1.370	74-73-65	119.5	64-66-67-69	175.8
73–74	1.741	69-67-68	112.6	61-60-58-57	−7.8
IT-2Br					
1–2	1.888	1-2-3	119.6	6-15-17-18	3.2
9–10	1.370	11-10-12	112.4	8-9-10-11	1.7
66–67	1.370	74-73-65	119.6	64-66-67-69	−177.6
73–74	1.888	69-67-68	112.4	61-60-58-57	3.2
PBDB-T					
2–3	1.463	1-2-3	126.5	1-2-3-4	130.2
6–7	1.463	5-6-7	128.5	5-6-7-8	−119.4
10–11	1.454	9-10-11	130.8	9-10-11-12	151.1
18–19	1.455	13-14-15	122.1	13-14-15-16	−126.7
22–23	1.459	18-19-20	130.1	59-60-61-62	57.5
14–15	1.473	21-22-23	128.6	17-18-19-20	−141.6
60–61	1.473	59-60-61	120.5	21-22-23-24	140.0

**Table 2:** The selected molecular orbital energies (in eV), HOMO-LUMO gap (H-Lgap, in eV) for the PBDB-T, ITIC and IT-2X (X = F, Cl, Br) molecules in solid phase. (LC-PBE/6-31G**; *α* = 0.200, *β* = 0.086; $\varepsilon_{s}$ = 3.5, $\varepsilon_{d}$ = 3.3; the ω unit is *Bohr*^−1^).

Molecule	HOMO−1	HOMO	LUMO	LUMO+1	H-L_gap_
PBDB-T	−5.53	−5.47	−2.31	−2.16	3.16
ITIC	−6.42	−5.72	−3.26	−3.04	2.46
IT-2F	−6.46	−5.75	−3.28	−3.04	2.48
IT-2Cl	−6.46	−5.76	−3.34	−3.12	2.42
IT-2Br	−6.47	−5.77	−3.34	−3.13	2.43

**Table 3:** The calculated transition energies (in eV), excitation wavelengths (in nm), excited states characters (ESC), corresponding oscillator strengths (*f*), and the main transition configurations with coefficients larger than 10%. CT and ACT represent the charge transfer from the donor segment to the acceptor segment and from the acceptor segment to the donor segment. DLE (ALE) represents the local excitation that occurs on the donor (acceptor) segment. The states with *f* > 0.1 and excitation wavelength longer than 400 nm are listed (LC-PBE/6-31G**; the ω unit is *Bohr*^−1^; *α* = 0.200, *β* = 0.086; $\varepsilon_{s}$ = 3.5, $\varepsilon_{d}$ = 3.3).

States	Main Transition Configurations	ESC	E (eV/nm)	*f*
PBDB-T (ω = 0.155)				
S1	H→L(83%)	CT&DLE	2.57/482.92	0.9508
S3	H-1→L + 2(27%); H→L + 2(29%); H→L + 3(13%)	CT	2.78/446.13	0.1666
S5	H-1→L + 2(11%); H→L + 1(11%)	CT	2.92/424.74	0.1366
	H→L + 2(23%); H→L + 3(32%)			
S7	H-2→L(62%)	CT&DLE	3.07/404.48	0.4274
ITIC (ω = 0.155)				
S1	H→L(96%)	CT&DLE	1.92/646.56	2.8141
S3	H→L + 2(91%)	CT&DLE	2.66/466.84	0.1901
S8	H-4→L(78%)	CT&DLE	2.92/424.18	0.3927
S9	H-1→L + 1(87%)	CT&DLE	2.95/420.57	0.1265
IT-2F (ω = 0.167)				
S1	H→L(96%)	CT&DLE	1.93/644.02	2.8684
S3	H→L + 2(90%)	CT&DLE	2.62/472.71	0.1270
S8	H-4→L(80%)	CT&DLE	2.93/423.68	0.3566
S9	H-1→L + 1(89%)	CT&DLE	2.97/417.18	0.1644
IT-2Cl (ω = 0.144)				
S1	H→L(96%)	CT&DLE	1.89/657.52	2.8394
S3	H→L + 2(91%)	CT&DLE	2.57/483.05	0.1742
S8	H-4→L(82%)	CT&DLE	2.87/432.15	0.3433
S9	H-1→L + 1(90%)	CT&DLE	2.91/426.22	0.1671
IT-2Br (ω = 0.155)				
S1	H→L(96%)	CT&DLE	1.89/657.37	2.8730
S3	H→L + 2(91%)	CT&DLE	2.58/480.80	0.1798
S8	H-4→L(79%)	CT&DLE	2.88/430.87	0.3450
S9	H-1→L + 1(90%)	CT&DLE	2.91/425.85	0.1808

**Table 4:** The selected dihedral angles (in °) of PBDB-T:ITIC and PBDB-T:IT-2X (X = F, Cl, Br) complexes. The corresponding atomic serial numbers are shown Figure S1 in Supporting Material. (ωB97XD/6-31G**).

Dihedral Angles	PBDB-T:ITIC	PBDB-T:IT-2F	PBDB-T:IT-2Cl	PBDB-T:IT-2Br
1-2-3-4 ^a^	126.4	130.0	149.3	131.0
5-6-7-8 ^a^	157.6	177.0	142.6	0.0
9-10-11-12 ^a^	−166.0	167.4	−150.6	174.5
59-60-61-62 ^a^	126.3	123.2	136.3	123.8
13-14-15-16 ^a^	−125.3	−62.9	−124.4	−56.1
17-18-19-20 ^a^	−136.5	131.2	−123.4	132.7
21-22-23-24 ^a^	128.0	−150.7	129.4	−152.7
25-26-27-28 ^a^	−159.9	−157.4	−163.4	−152.8
29-30-31-32 ^a^	155.1	158.0	−178.5	−171.1
33-34-35-36 ^a^	−116.0	−117.2	−115.5	−111.7
37-38-39-40 ^a^	−142.3	−145.1	−131.2	−139.6
6-15-17-18 ^b^	−1.1	−19.0	−2.5	−11.9
37-21-30-31 ^b^	101.2	87.0	−73.9	−84.5
20-21-22-23 ^b^	93.6	103.4	91.3	105.9
42-41-50-51 ^b^	−18.6	−21.3	−15.6	−19.6
40-41-43-44 ^b^	−4.1	−4.3	−8.6	−4.6
61-60-58-57 ^b^	−11.0	−8.2	9.2	12.8

^a^ the atomic serial numbers of the PBDB-T. ^b^ the atomic serial numbers of the electron acceptors.

**Table 5:** The HOMO and LUMO energies (in eV), HOMO−LUMO Gaps (H−L_gap_, in eV), and binding energies E_b_ (in eV) of the complexes formed by donor and acceptor. (LC-PBE/6-31G**; *α* = 0.200, *β* = 0.086; *ε_s_* = 3.5, *ε_d_* = 3.3; the ω unit is *Bohr*^−1^).

Complexes	ω	HOMO	LUMO	H-L_gap_	E_b_
PBDB-T:ITIC	0.155	−5.34	−3.12	2.23	3.34
PBDB-T:IT-2F	0.167	−5.40	−3.10	2.30	3.62
PBDB-T:IT-2Cl	0.144	−5.34	−3.24	2.09	3.35
PBDB-T:IT-2Br	0.155	−5.37	−3.16	2.21	3.80

**Table 6:** Electronic transition energies (in eV), corresponding oscillator strengths (*f*), excitation wavelengths (in nm), excited states characters (ESC), and main transition configurations with coefficients larger than 10% for the selected excited states of PBDB-T:ITIC and PBDB-T:IT-2X (X = F, Cl, Br) complexes. (LC-PBE/6-31G**, the ω unit is *Bohr*^−1^; *α* = 0.200, *β* = 0.086; *ε_s_* = 3.5, ε_d_ = 3.3).

States	Main Transitioncon Figurations	ESC	E (eV/nm)	*f*
PBDB-T:ITIC (ω = 0.155)				
S1	H→L(75%); H→L + 1(16%)	CT	1.60/777.01	0.0522
S3	H-2→L(75%); H→L + 1(13%)	ALE	1.95/636.26	1.9895
S9	H-2→L + 1(59%)	ALE	2.30/539.93	0.1396
S11	H→L + 3(47%); H→L + 4(22%)	CT&DLE	2.38/521.46	1.4261
PBDB-T:IT-2F (ω = 0.167)				
S1	H-1→L(21%); H-1→L + 1(14%); H→L(60%)	CT	1.71/723.8	0.0398
S3	H-2→L(88%)	ALE	1.91/647.97	2.1042
S11	H-1→L + 5(12%); H→L + 3(10%); H→L + 4(46%)	DLE	2.43/509.86	1.5504
PBDB-T:IT-2Cl (ω = 0.144)				
S1	H→L(79%); H→L + 1(16%)	CT	1.47/844.09	0.0212
S3	H-2→L(89%)	ALE	1.89/654.39	2.1403
S12	H→L + 4(23%); H→L + 5(62%)	DLE	2.42/513.06	1.2822
PBDB-T:IT-2Br (ω = 0.155)				
S1	H→L(75%)	CT	1.62/767.38	0.0266
S3	H-2→L(90%)	ALE	1.87/662.95	2.0919
S11	H→L + 3(11%); H→L + 4(62%)	DLE	2.39/518.12	1.4911

**Table 7:** Calculated reorganization energies λ (in eV), free energy changes ΔG (in eV), electron coupling V (in eV) and rate constants K (in s^−1^) of the PBDB-T:ITIC and PBDB-T:IT-2X (X = F, Cl, Br) complexes for the three electron processes.

Acceptors	ETP	λ_i_	λ_ext_	λ	∆G	V	|∆G + λ|	K
ITIC	CT	0.179	0.024	0.203	−0.811	0.179	0.608	2.69 × 10^7^
	CR	0.182	0.024	0.206	−1.250	0.369	1.044	3.26 × 10^−7^
	ED	0.157	0.024	0.181	−0.545	0.038	0.364	4.81 × 10^10^
IT-2F	CT	0.182	0.023	0.205	−0.772	0.153	0.567	2.29 × 10^8^
	CR	0.186	0.023	0.209	−1.258	0.329	1.049	2.73 × 10^−7^
	ED	0.164	0.023	0.187	−0.537	0.018	0.350	2.16 × 10^10^
IT-2Cl	CT	0.181	0.026	0.207	−0.805	0.053	0.598	5.49 × 10^6^
	CR	0.183	0.026	0.208	−1.171	0.178	0.963	2.56 × 10^−4^
	ED	0.165	0.026	0.190	−0.603	0.060	0.413	2.38 × 10^10^
IT-2Br	CT	0.176	0.023	0.199	−0.776	0.047	0.577	8.31 × 10^6^
	CR	0.180	0.023	0.202	−1.193	0.261	0.991	1.09 × 10^−5^
	ED	0.164	0.023	0.187	−0.484	0.026	0.297	2.70 × 10^11^

## Data Availability

The data presented in this study are available on request from the corresponding author.

## Supplementary Materials

The following are available online at https://www.mdpi.com/article/10.3390/nano11123417/s1. Figure S1: The optimized structures of PBDB-T, ITIC and IT-2X (X = F, Cl, Br) in gas phase. The atomic serial numbers are also labeled to give geometrical parameters. The hydrogen atoms are omitted for clarity. (ωB97XD/6-31G**). Figure S2: The selected frontier molecular orbitals involved in transition configurations for the ITIC and IT-2X (X = F, Cl, Br) and PBDB-T. (LC-PBE/6-31G**; ω = 0.155, 0.167, 0.144, 0.155, 0.155 *Bohr*^−1^; *α* = 0.200, *β* = 0.086; *ε_s_* = 3.5, *ε_d_* = 3.3; H = HOMO, L = LUMO). Figure S3: Charge density difference for the low-lying excited states of ITIC, IT-2X (X = F, Cl, Br) and PBDB-T molecules. The regions colored in red (blue) indicate the increase (decrease) of electron density during excitation processes, respectively. The charge transfer distance Δd and the transferred charges Δq are shown. (LC-PBE/6-31G**; ω = 0.155, 0.167, 0.144, 0.155 *Bohr*^−1^; *α* = 0.200, *β* = 0.086; *ε_s_* = 3.5, *ε_d_* = 3.3). Figure S4: Selected frontier molecular orbitals for PBDB-T:ITIC and PBDB-T:IT-2X (X = F, Cl, Br) complexes. (LC-PBE/6-31G**; ω = 0.155, 0.167, 0.144, 0.155 *Bohr*^−1^; *α* = 0.200, *β* = 0.086; *ε_s_* = 3.5, *ε_d_* = 3.3; H = HOMO, L = LUMO). Figure S5: Charge density difference for the low-lying excited states of PBDB-T:ITIC and PBDB-T:IT-2X (X = F, Cl, Br) complexes. The regions colored in red (blue) indicate the increase (decrease) of electron density during excitation processes, respectively. The charge transfer distance Δd and the transferred charges Δq are shown. (LC-PBE/6-31G**; ω = 0.155, 0.167, 0.144, 0.155 *Bohr*^−1^; *α* = 0.200, *β* = 0.086; *ε_s_* = 3.5, *ε_d_* = 3.3), Table S1: The selected geometrical parameters, including bond lengths (in Å), bond angles (in°) and dihedral angles (in°) which were defined by two, three and four atoms, respectively. (ωB97XD/6-31G**). Table S2: Electronic transition energies (in eV), excitation wavelengths (in nm), excited states characters (ESC), corresponding oscillator strengths (*f*), and the main transition configurations with coefficients larger than 10% for the PBDB-T, ITIC and IT-2X (X = F, Cl, Br). CT (ACT) represents the charge transfer from the donor (acceptor) segment to the acceptor (donor) segment. DLE (ALE) represents the local excitation that occurs on the donor (acceptor) segment. (LC-PBE/6-31G**; *α* = 0.200, *β* = 0.086; *ε_s_* = 3.5, *ε_d_* = 3.3; ω unit is *Bohr*^−1^). Table S3: The selected bond lengths (in Å) and bond angles (in °) of PBDB-T:ITIC and PBDB-T:IT-2X (X = F, Cl, Br) complexes. The atomic serial numbers is shown in Figure S1 of supporting material. (ωB97XD/6-31G**). Table S4: Electronic transition energies (in eV), corresponding oscillator strengths (*f*), excitation wavelengths (in nm), excited states characters (ESC) and main transition configurations with coefficients larger than 10% for PBDB-T:ITIC and PBDB-T:IT-2X (X = F, Cl, Br) complexes. (LC-PBE/6-31G**; *ε_s_* = 3.5, *ε_d_* = 3.3; α = 0.200, β = 0.086; the ω unit is *Bohr*^−1^). Table S5: The components of the dipole moment (in Debye) and quadrupole moment (in Debye-Ang) of ITIC and IT-2X (X = F, Cl, Br) molecules. ($U_{1}=\sqrt{X^{2}+Y^{2}+Z^{2}}$; LC-PBE/6-31G**; ω = 0.155, 0.167, 0.144, 0.155 *Bohr*^−1^; *α* = 0.200, *β* = 0.086; *ε_s_* = 3.5, *ε_d_* = 3.3). Table S6: Calculated electronic coupling V (in eV) in CR process of PBDB-T:ITIC and PBDB-T:IT-2X (X = F, Cl, Br) complexes. u correspond the transition dipole moment (in a.u); μ_g_ and μ_e_ represent the dipole moment at the ground state and excited state (in Debye), respectively. Δμ_ge_ indicates the change in dipole moment between the ground state and the lowest singlet excited state (in Debye); ΔE is the energy differnence between the ground state and the excited state (in eV). (LC-PBE/6-31G**; ω = 0.155, 0.167, 0.144, 0.155 *Bohr*^−1^; *α* = 0.200, *β* = 0.086; *ε_s_* = 3.5, *ε_d_* = 3.3). Table S7: Electronic coupling V (in meV) in CT and ED process of PBDB-T:ITIC and PBDB-T:IT-2X (X = F, Cl, Br) complexes. E_i_ and E_f_ are the energy (in eV) of initial and final states; S_if_ covresponds the overlap matrix element, and H_if_ (meV) is the off-diagonal hamiltonian of charge-localized state. (LC-PBE/6-31G**; ω = 0.155, 0.167, 0.144, 0.155 *Bohr*^−1^; *α* = 0.200, *β* = 0.086; *ε_s_* = 3.5, *ε_d_* = 3.3).
