# Supplementary material for: The Halogenation Effects of Electron Acceptor ITIC for Organic Photovoltaic Nano-Heterojunctions
Source: Nanomaterials (Basel). 2021 Dec 16;11(12):3417. doi: 10.3390/nano11123417 (PMC8708652; doi:10.3390/nano11123417)
Supplement: Supplementary file 1 [file nanomaterials-11-03417-s001.zip › nanomaterials-1457559-supplementary.pdf]

Supplementary material

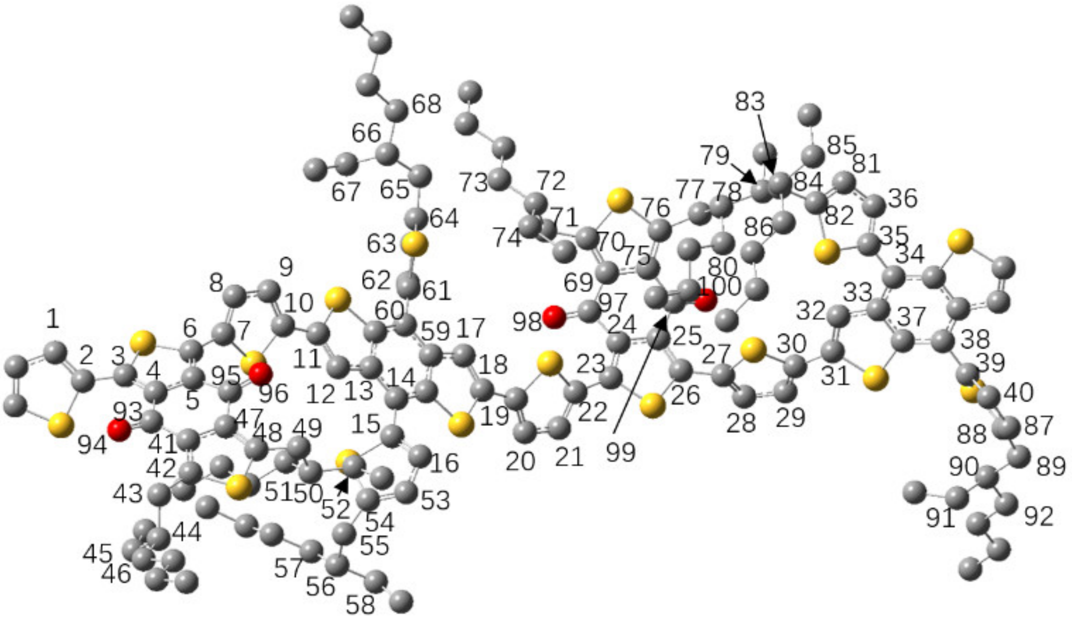

PBDB-T

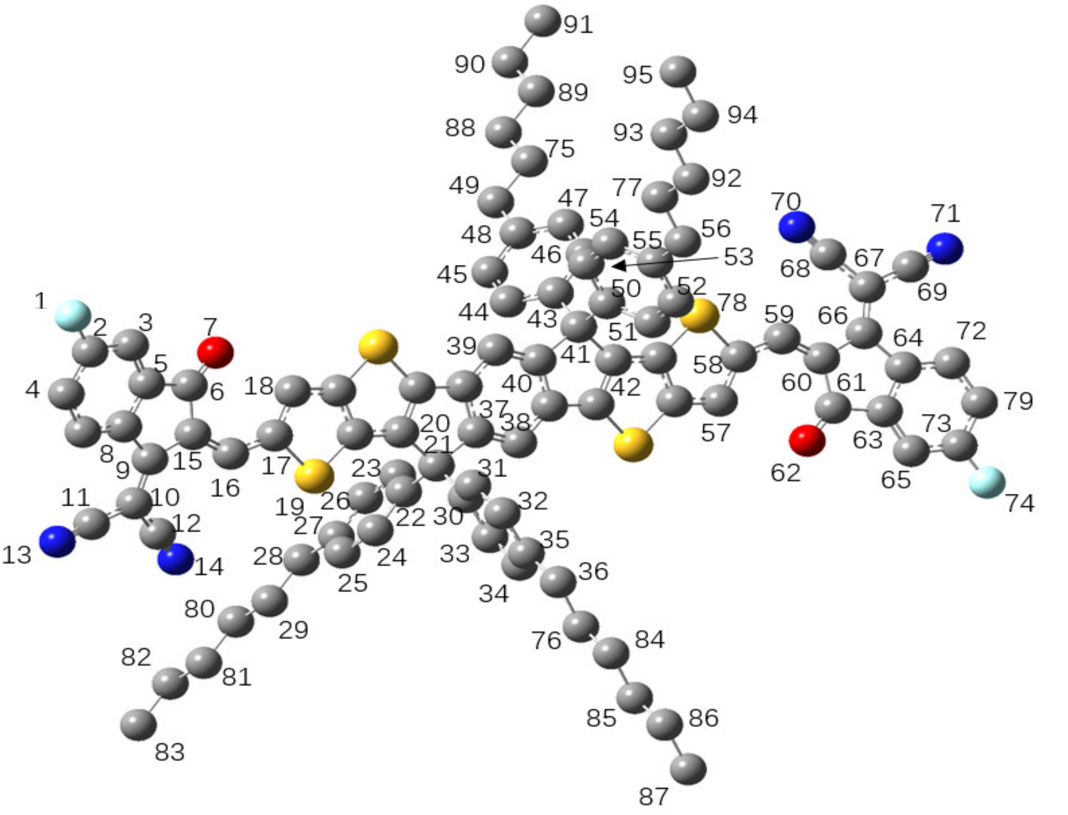

ITIC and IT-2X (X= F, Cl, Br)

Figure S1. The optimized structures of PBDB-T, ITIC and IT-2X (X=F, Cl, Br) in gas phase. The atomic serial numbers are also labeled to give geometrical parameters. The hydrogen atoms are omitted for clarity. (ωB97XD/6-31G**).

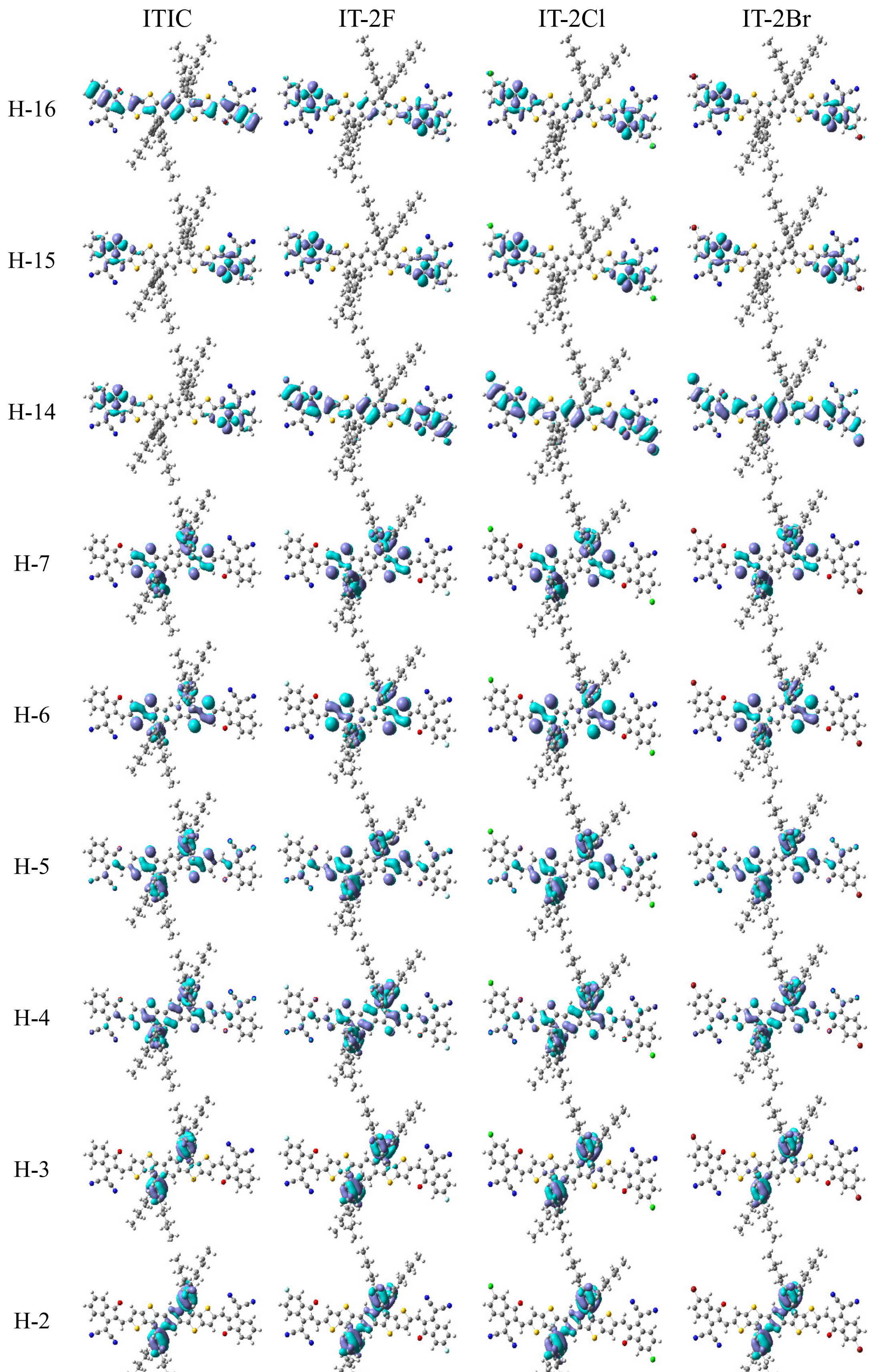
ITIC
IT-2F
IT-2Cl
IT-2Br
H-16
H-15
H-14
H-7
H-6
H-5
H-4
H-3
H-2

H-1
H
L
L+1
L+2
L+3
L+4
L+5

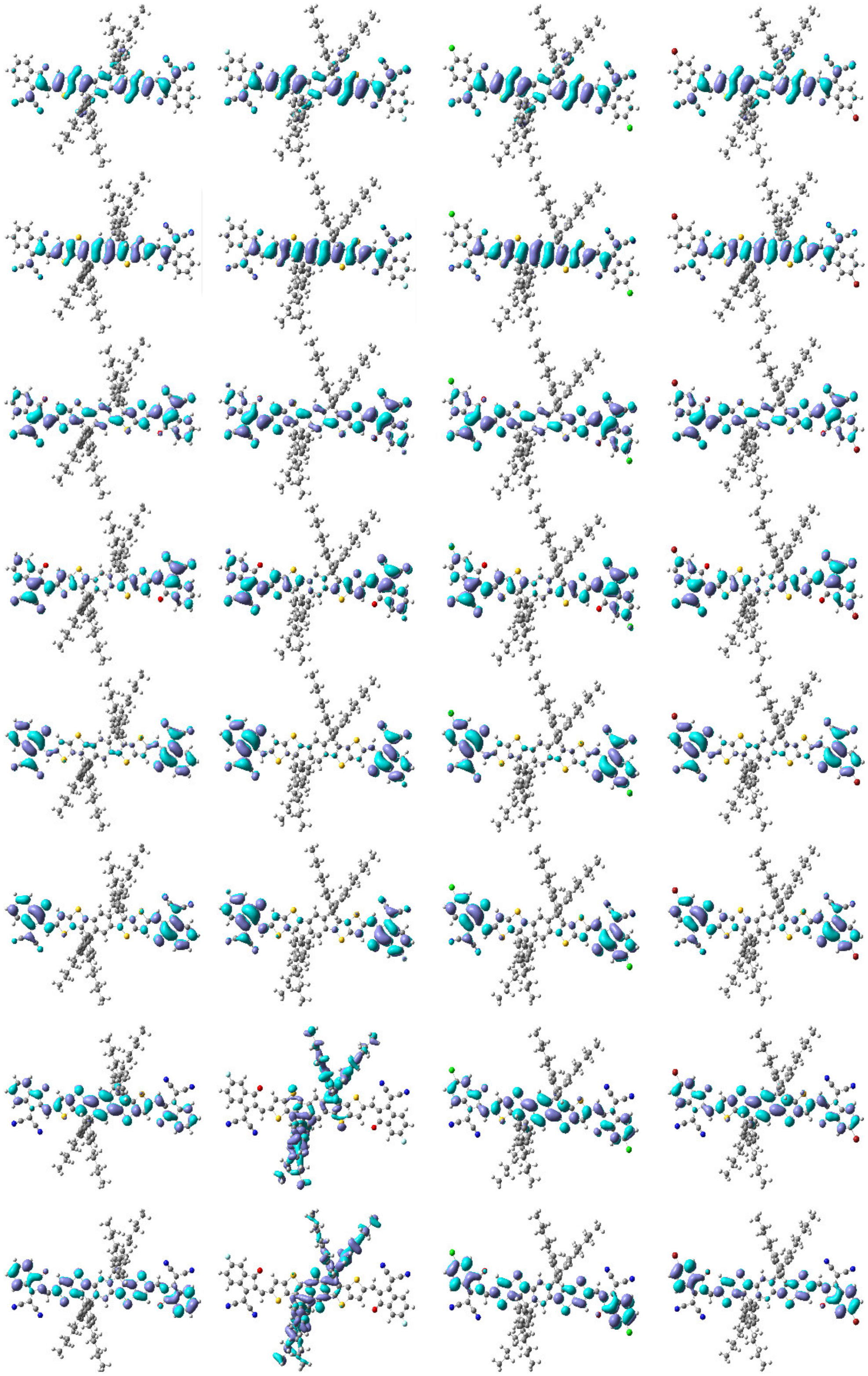

PBDB-T

H

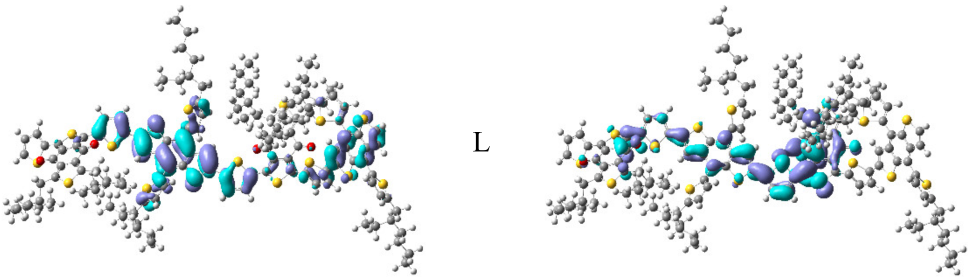

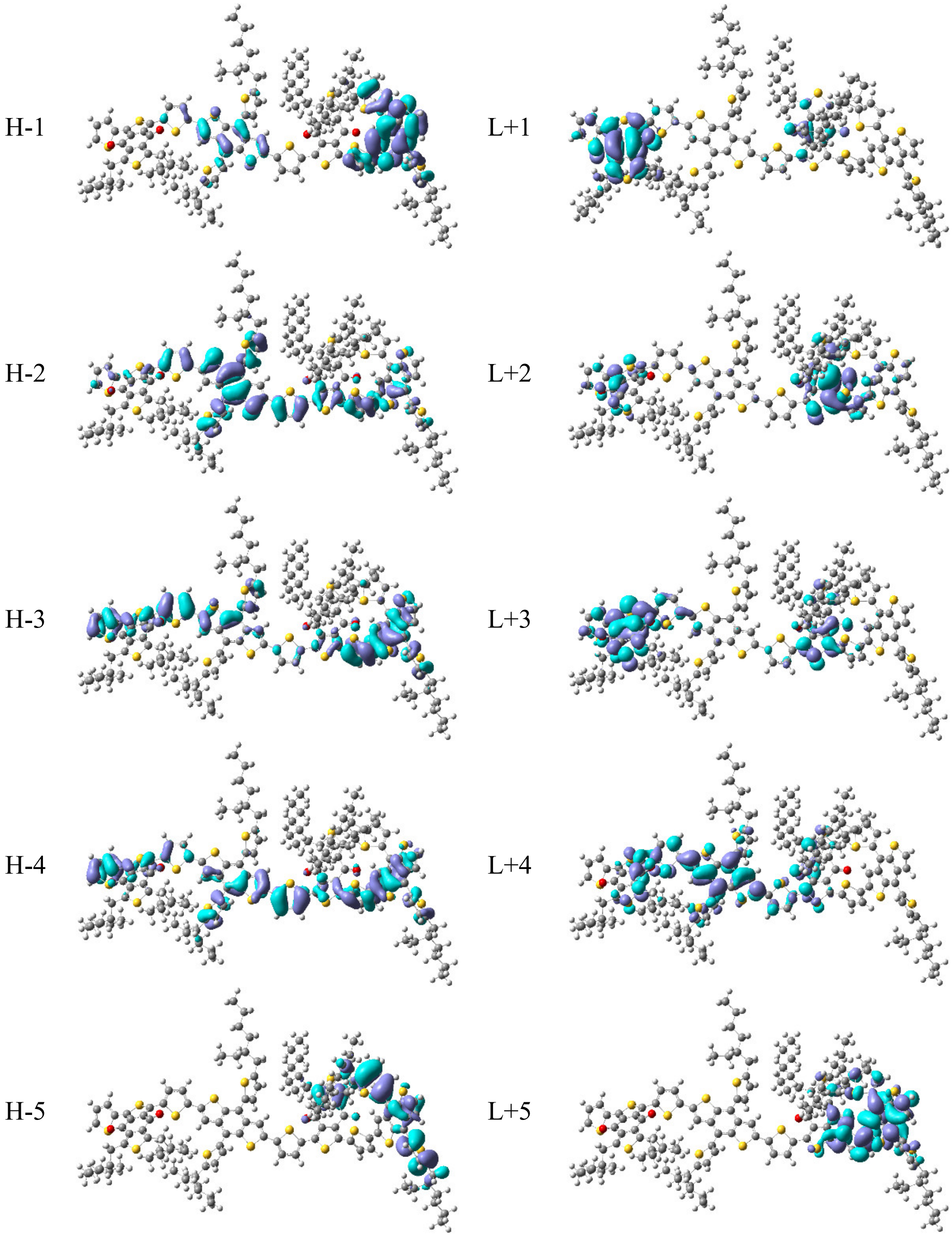

Figure S2. The selected frontier molecular orbitals involved in transition configurations for the ITIC and IT-2X (X= F, Cl, Br) and PBDB-T. (LC-PBE/6-31G**; ω=0.155, 0.167, 0.144, 0.155, 0.155 *Bohr*$^{-1}$; α=0.200, β=0.086; ε_s=3.5,ε_d=3.3; H=HOMO, L=LUMO).

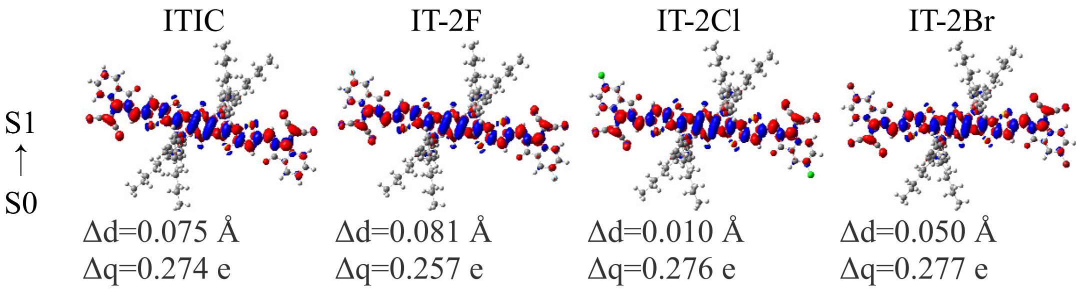

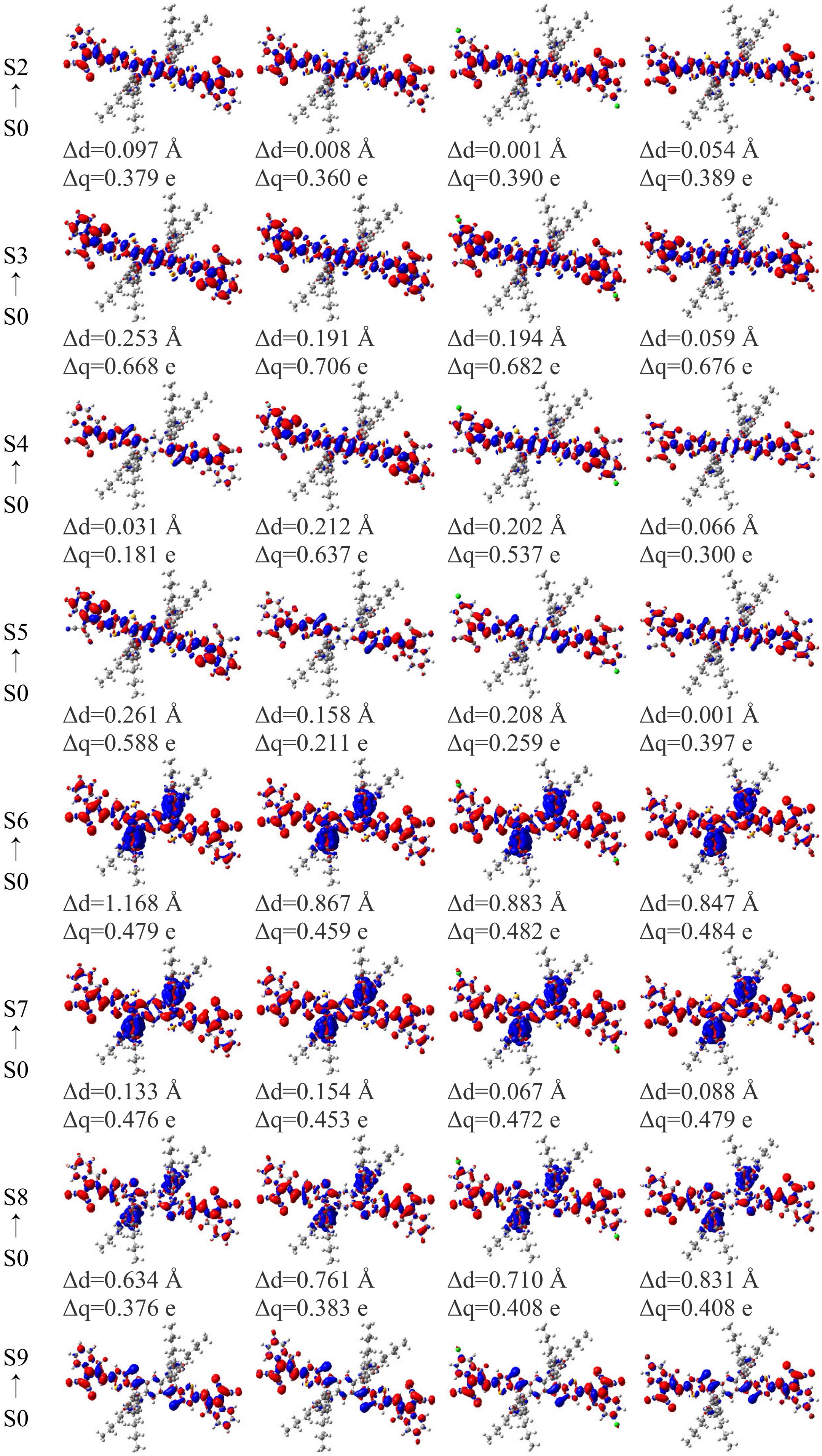
S2 ↑ S0
Δd=0.097 Å
Δq=0.379 e
Δd=0.008 Å
Δq=0.360 e
Δd=0.001 Å
Δq=0.390 e
Δd=0.054 Å
Δq=0.389 e
S3 ↑ S0
Δd=0.253 Å
Δq=0.668 e
Δd=0.191 Å
Δq=0.706 e
Δd=0.194 Å
Δq=0.682 e
Δd=0.059 Å
Δq=0.676 e
S4 ↑ S0
Δd=0.031 Å
Δq=0.181 e
Δd=0.212 Å
Δq=0.637 e
Δd=0.202 Å
Δq=0.537 e
Δd=0.066 Å
Δq=0.300 e
S5 ↑ S0
Δd=0.261 Å
Δq=0.588 e
Δd=0.158 Å
Δq=0.211 e
Δd=0.208 Å
Δq=0.259 e
Δd=0.001 Å
Δq=0.397 e
S6 ↑ S0
Δd=1.168 Å
Δq=0.479 e
Δd=0.867 Å
Δq=0.459 e
Δd=0.883 Å
Δq=0.482 e
Δd=0.847 Å
Δq=0.484 e
S7 ↑ S0
Δd=0.133 Å
Δq=0.476 e
Δd=0.154 Å
Δq=0.453 e
Δd=0.067 Å
Δq=0.472 e
Δd=0.088 Å
Δq=0.479 e
S8 ↑ S0
Δd=0.634 Å
Δq=0.376 e
Δd=0.761 Å
Δq=0.383 e
Δd=0.710 Å
Δq=0.408 e
Δd=0.831 Å
Δq=0.408 e
S9 ↑ S0

Δd=0.020 Å Δq=0.293 e | Δd=0.145 Å Δq=0.282 e | Δd=0.167 Å Δq=0.315 e | Δd=0.161 Å Δq=0.313 e

S10 ↑ S0

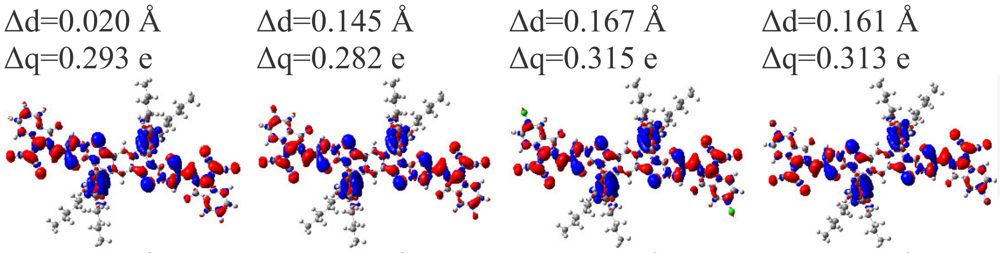

Δd=0.361 Å Δq=0.397 e | Δd=0.087 Å Δq=0.367 e | Δd=0.312 Å Δq=0.398 e | Δd=0.209 Å Δq=0.394 e

PBDB-T

S1 ↑ S0

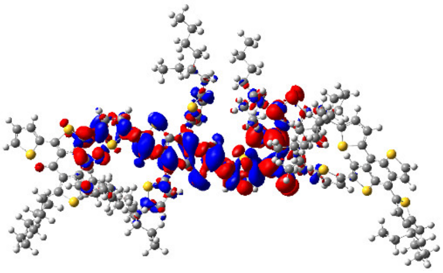

Δd=3.916 Å
Δq=0.584 e

S6 ↑ S0

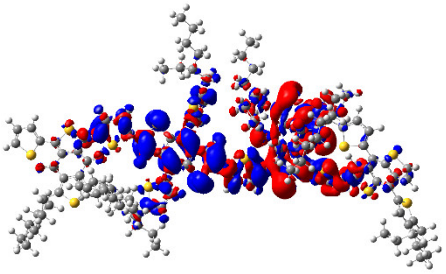

Δd=8.130 Å
Δq=0.787 e

S2 ↑ S0

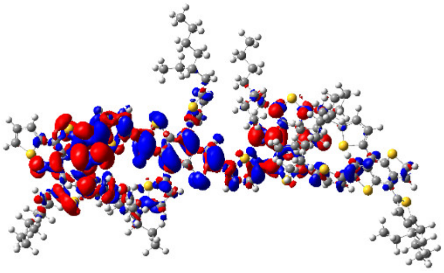

Δd=3.930 Å
Δq=0.807 e

S7 ↑ S0

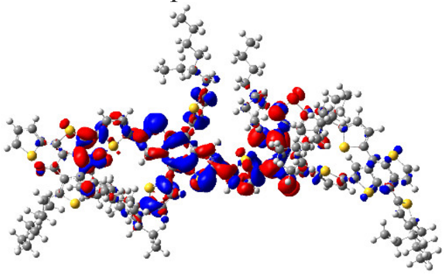

Δd=1.356 Å
Δq=0.584 e

S3 ↑ S0

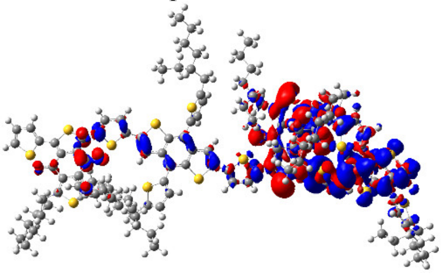

Δd=4.463 Å
Δq=0.771 e

S8 ↑ S0

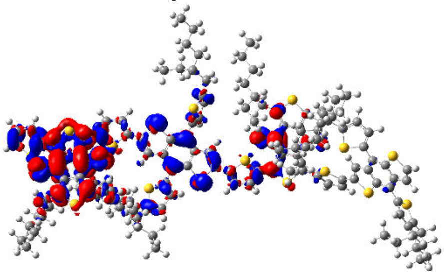

Δd=3.224 Å
Δq=0.619 e

S4 ↑ S0

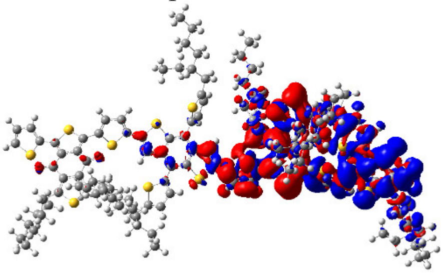

Δd=8.432 Å
Δq=0.659 e

S9 ↑ S0

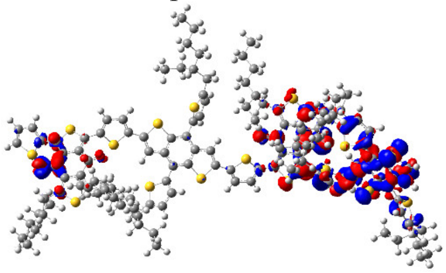

Δd=2.440 Å
Δq=0.378 e

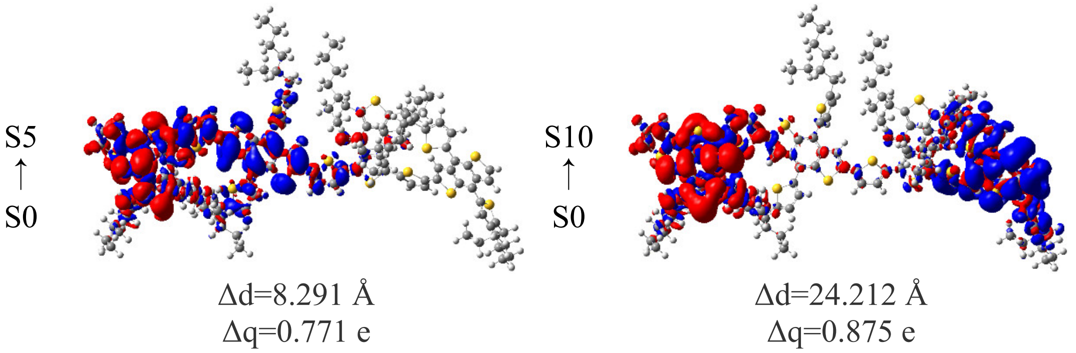

Figure S3. Charge density difference for the low-lying excited states of ITIC, IT-2X (X= F, Cl, Br) and PBDB-T molecules. The regions colored in red (blue) indicate the increase (decrease) of electron density during excitation processes, respectively. The charge transfer distance △d and the transferred charges △q are shown. (LC-PBE/6-31G**; ω=0.155, 0.167, 0.144, 0.155 *Bohr*$^{-1}$; α=0.200, β=0.086; ε_s=3.5,ε_d=3.3).

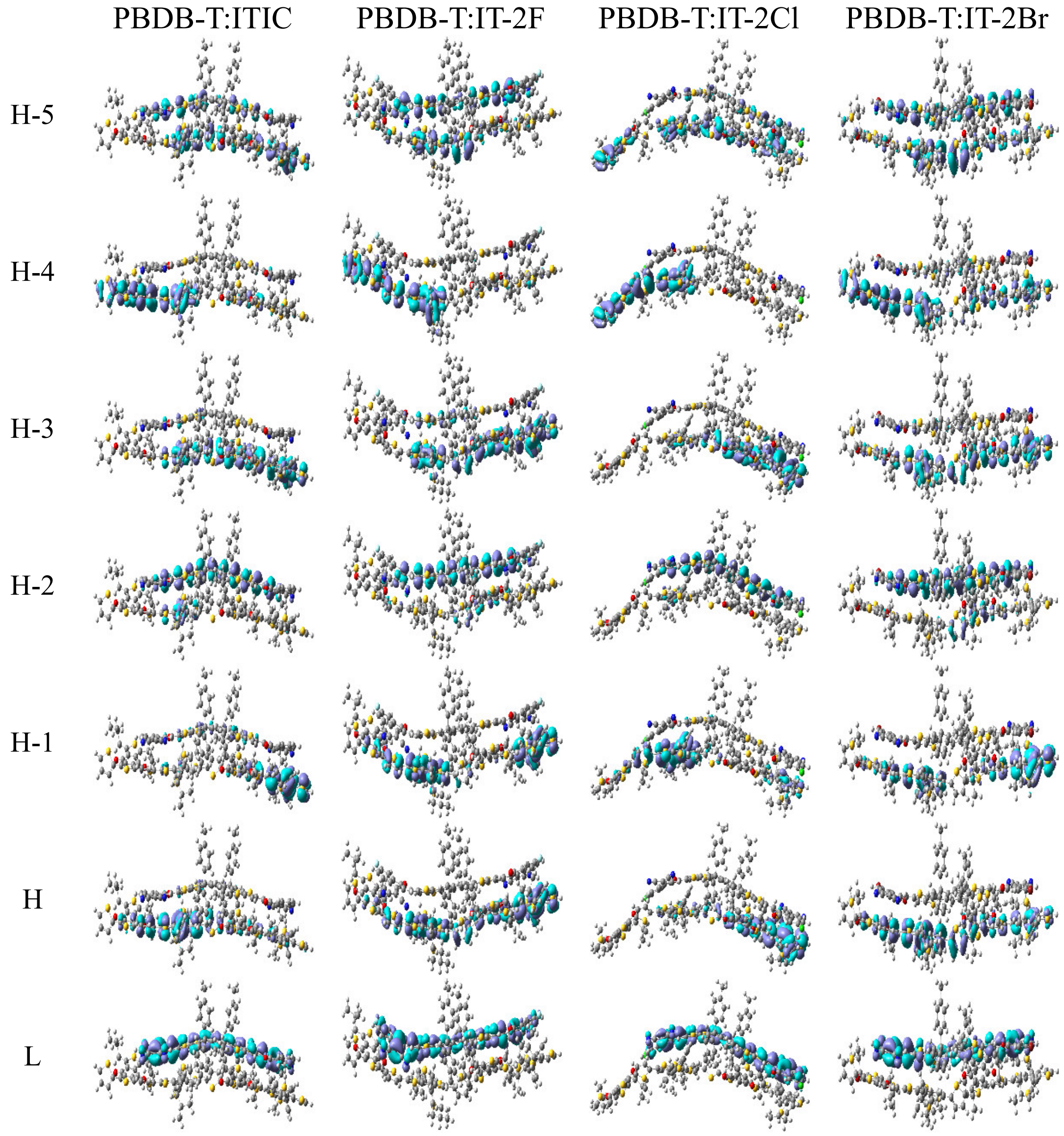

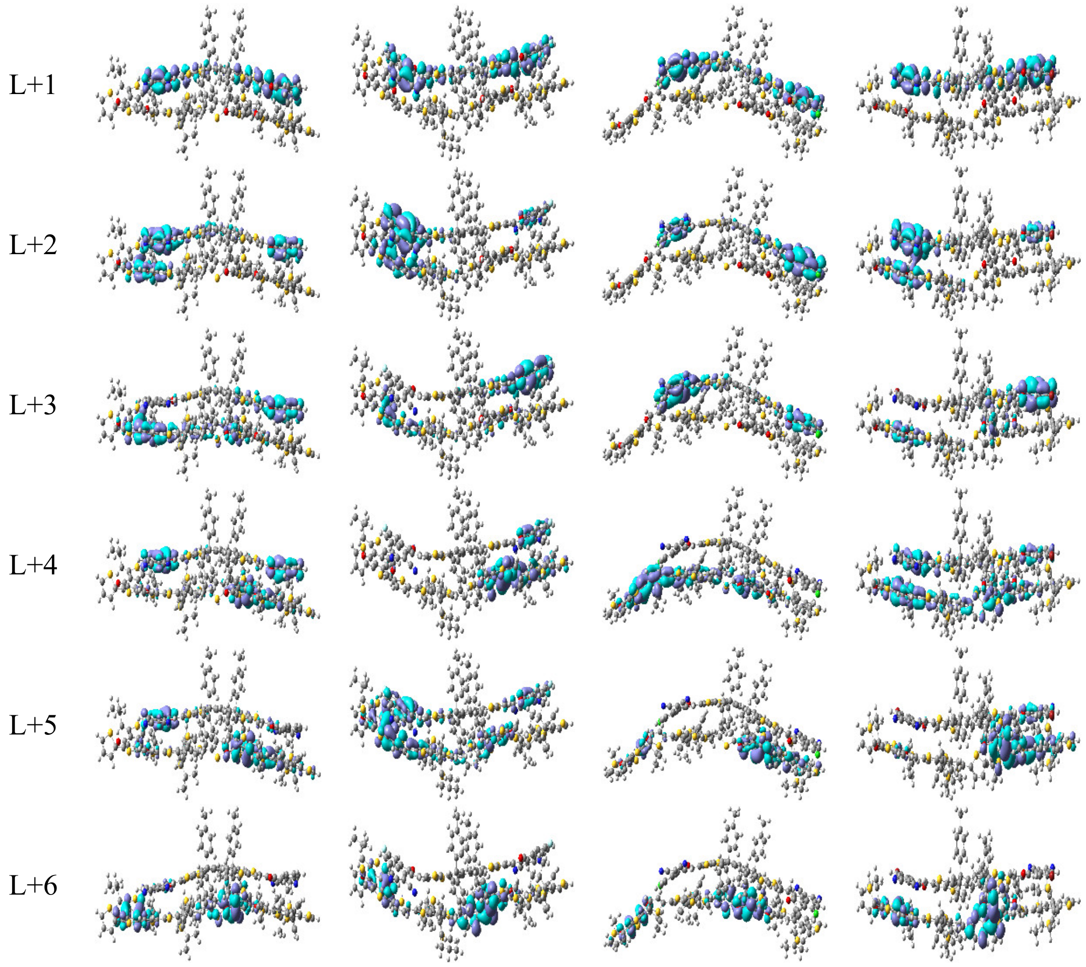

Figure S4. Selected frontier molecular orbitals for PBDB-T:ITIC and PBDB-T:IT-2X (X= F, Cl, Br) complexes. (LC-PBE/6-31G**; ω=0.155, 0.167, 0.144, 0.155 $Bohr^{-1}$; α=0.200, β=0.086; ε_s=3.5,ε_d=3.3; H=HOMO, L=LUMO).

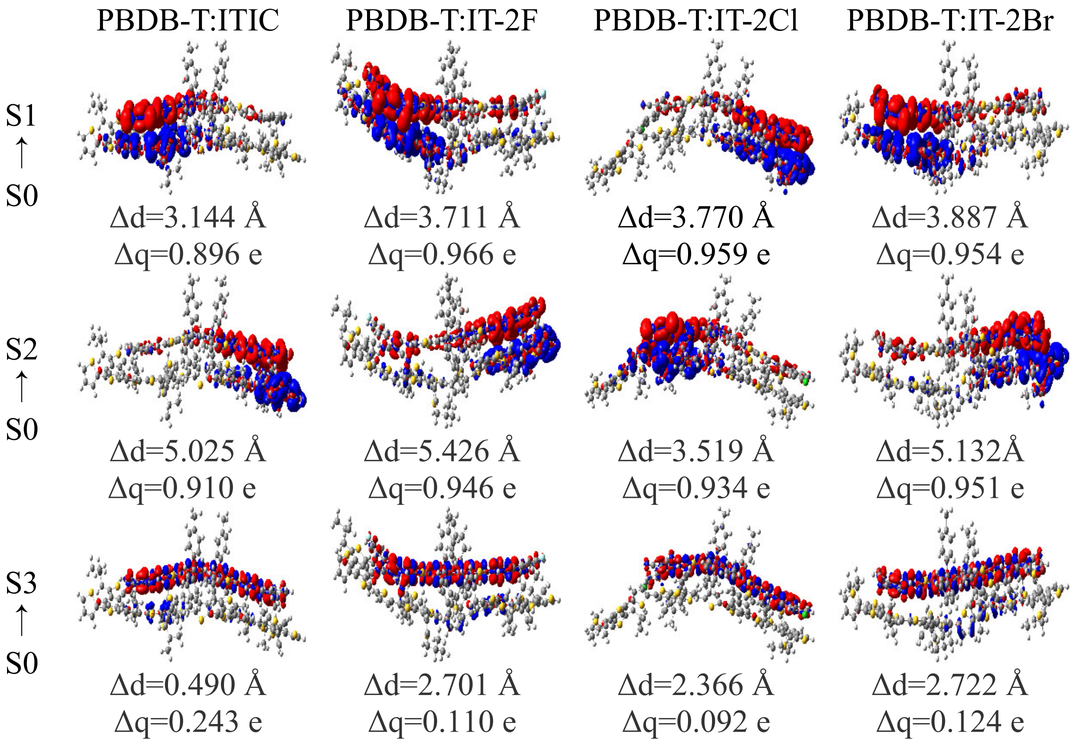

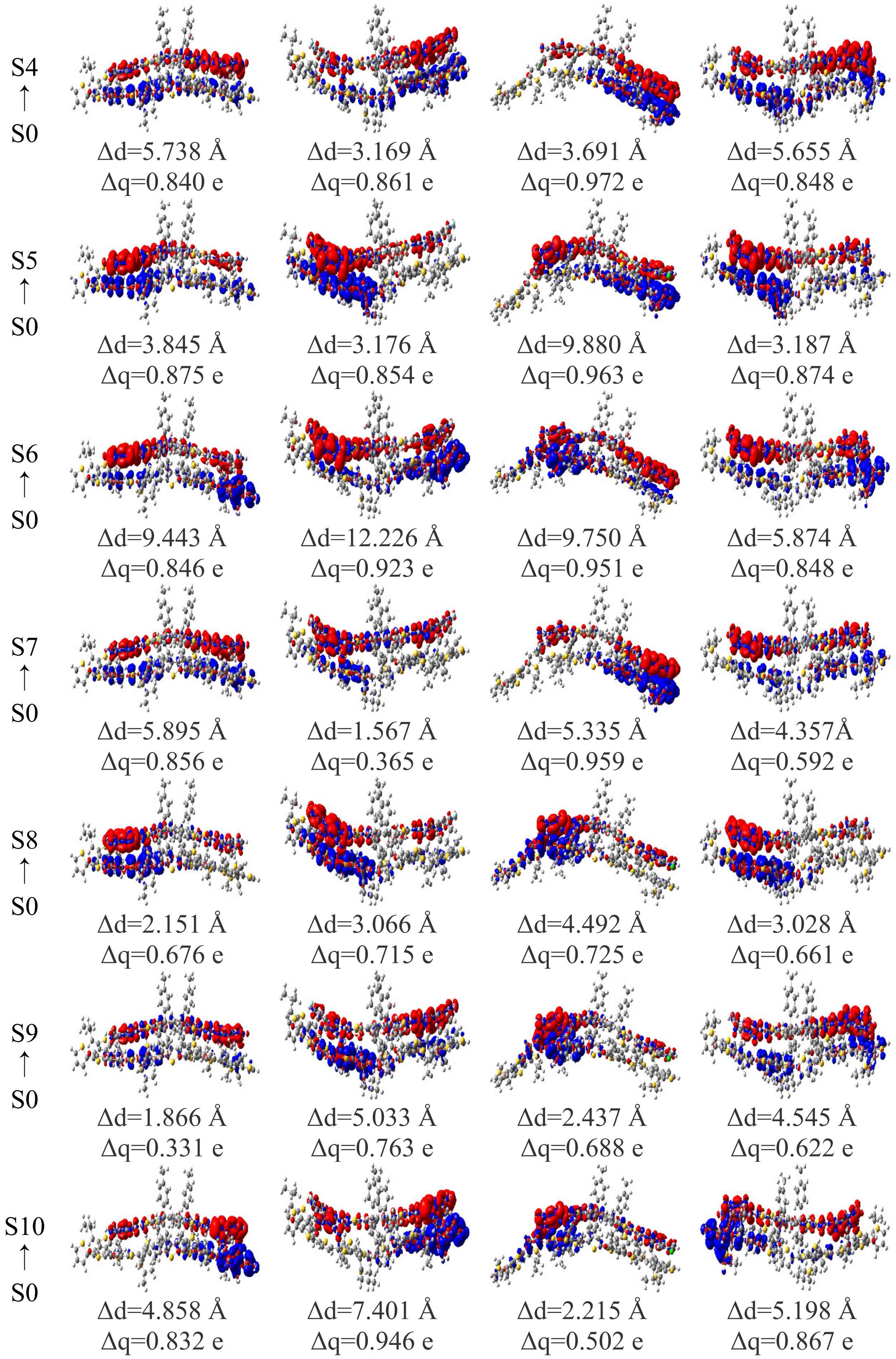

Figure S5. Charge density difference for the low-lying excited states of PBDB-T:ITIC and PBDB-T:IT-2X (X= F, Cl, Br) complexes. The regions colored in red (blue) indicate the increase (decrease) of electron density during excitation processes, respectively. The charge transfer distance △d and the transferred charges △q are shown. (LC-PBE/6-31G**; ω=0.155, 0.167, 0.144, 0.155 *Bohr*$^{-1}$; α=0.200, β=0.086; ε_s=3.5,ε_d=3.3).

Full name

ITIC: 3,9-bis(2-methylene-(3-(1,1-dicyanomethylene)-indanone))-5,5,11,11-tetrakis(4-hexylphenyl)-dithieno[2,3-d:2',3'd']-s-indaceno[1,2-b:5,6-b']-dithiophene

PBDB-T: poly[2,6-(4,8-bis(5-(2-ethylhexyl)thiophen-2-yl)benzo[1,2-b:4,5-b'] dithiophene)-co(1,3-di(5-thiophene-2-yl)-5,7-bis(2-ethylhexyl)-benzo[1,2-c:4,5-c'] dithiophene-4,8dione)]

Table S1. The selected geometrical parameters, including bond lengths (in Å), bond angles (in°) and dihedral angles (in°) which were defined by two, three and four atoms, respectively. (ωB97XD/6-31G**).

Definition	Bond length	Definition	Bond angles	Definition	Dihedral angles
			ITIC		
1-2	1.085	1-2-3	120.1	8-9-10-11	1.8
6-7	1.218	7-6-15	119.9	9-10-11-13	177.4
9-10	1.370	8-9-10	124.4	9-10-12-14	178.7
10-11	1.432	9-10-11	124.3	11-13-12-14	179.8
10-12	1.432	9-10-12	124.3	5-3-2-1	-179.8
11-13	1.160	10-11-13	177.7	15-9-10-12	0.5
12-14	1.161	10-12-14	178.0	6-15-17-18	3.8
15-16	1.368	11-10-12	112.2	9-15-17-19	1.8
16-17	1.428	6-15-16	112.4	20-21-30-31	-19.3
21-22	1.535	15-16-17	133.1	37-21-30-31	90.6
27-28	1.515	16-17-18	133.1	32-35-36-76	-179.3
28-29	1.526	20-21-22	108.4	35-36-76-84	179.8
29-80	1.528	20-21-30	112.8	36-76-84-85	0
80-81	1.528	37-21-22	113.4	76-84-85-86	-179.9
21-30	1.534	37-21-30	108.6	20-21-22-23	89.2
35-36	1.515	21-22-23	121.6	37-21-22-23	-20.8
36-76	1.526	28-27-26	119.1	26-27-28-29	179.6
76-84	1.528	21-30-31	121.3	27-28-29-80	-179.1
84-85	1.528	36-35-32	119.1	28-29-80-81	179.9
41-43	1.535	42-41-50	112.8	29-80-81-82	-179.4
48-49	1.515	42-41-43	108.4	40-41-50-51	90.6
49-75	1.526	40-41-50	108.6	42-41-50-51	-19.3
75-88	1.528	40-41-43	113.4	52-55-56-77	-179.3
88-89	1.528	41-50-51	121.3	55-56-77-92	179.8
41-50	1.534	56-55-52	119.1	56-77-92-93	0
55-56	1.515	41-43-44	121.6	77-92-93-94	-179.9
56-77	1.526	49-48-45	119.1	40-41-43-44	-20.8
77-92	1.528	74-73-65	120.1	42-41-43-44	89.2
92-93	1.528	62-61-60	129.0	45-48-49-75	-179.5
58-59	1.428	64-66-67	124.1	48-49-75-88	-179.1
59-60	1.368	66-67-69	123.3	49-75-88-89	179.9
61-62	1.218	66-67-68	124.6	75-88-89-90	-179.4
66-67	1.370	69-67-68	112.2	64-66-67-69	-177.5
67-68	1.432	67-69-71	177.7	66-67-69-71	177.4
67-69	1.432	67-68-70	178.0	66-67-68-70	178.7
68-70	1.161	61-60-59	112.2	69-71-68-70	179.8
69-71	1.160	61-60-59	127.7	63-65-73-74	-179.8
73-74	1.085	60-59-58	133.1	64-66-67-68	1.8
82-83	1.527	57-58-59	133.1	61-60-58-57	3.8
86-87	1.527	22-21-30	113.1	66-60-58-78	1.8
90-91	1.527	43-41-50	113.1		

94-95	1.527				

Definition	Bond length	Definition	Bond angles	Definition	Dihedral angles
			IT-2F		
1-2	1.335	1-2-3	119.2	8-9-10-11	-2.6
6-7	1.217	7-6-15	121.6	9-10-11-13	-174.8
9-10	1.370	8-9-10	124.5	9-10-12-14	-175.5
10-11	1.432	9-10-11	123.0	11-13-12-14	-179.6
10-12	1.432	9-10-12	124.4	5-3-2-1	179.8
11-13	1.160	10-11-13	178.2	15-9-10-12	-1.1
12-14	1.161	10-12-14	178.2	6-15-17-18	-7.6
15-16	1.369	11-10-12	112.6	9-15-17-19	-3.9
16-17	1.427	6-15-16	127.7	20-21-30-31	-20.3
21-22	1.534	15-16-17	132.9	37-21-30-31	89.4
27-28	1.515	16-17-18	132.9	32-35-36-76	176.9
28-29	1.527	20-21-22	108.3	35-36-76-84	-178.8
29-80	1.528	20-21-30	112.8	36-76-84-85	-179.7
80-81	1.528	37-21-22	113.6	76-84-85-86	-179.7
21-30	1.534	37-21-30	108.4	20-21-22-23	89.9
35-36	1.515	21-22-23	121.7	37-21-22-23	-20.2
36-76	1.527	28-27-26	119.1	26-27-28-29	-177.5
76-84	1.528	21-30-31	121.2	27-28-29-80	179.9
84-85	1.528	36-35-32	119.1	28-29-80-81	179.8
41-43	1.534	42-41-50	112.8	29-80-81-82	-179.4
48-49	1.515	42-41-43	108.3	40-41-50-51	89.4
49-75	1.527	40-41-50	108.4	42-41-50-51	-20.3
75-88	1.528	40-41-43	113.6	52-55-56-77	176.9
88-89	1.528	41-50-51	121.3	55-56-77-92	-178.8
41-50	1.534	56-55-52	119.1	56-77-92-93	-179.7
55-56	1.515	41-43-44	121.7	77-92-93-94	-179.6
56-77	1.527	49-48-45	119.8	40-41-43-44	-20.2
77-92	1.528	74-73-65	119.2	42-41-43-44	89.9
92-93	1.528	62-61-60	129.4	45-48-49-75	-177.5
58-59	1.427	64-66-67	124.5	48-49-75-88	179.9
59-60	1.369	66-67-69	123.0	49-75-88-89	179.8
61-62	1.217	66-67-68	124.4	75-88-89-90	-179.4
66-67	1.370	69-67-68	112.6	64-66-67-69	176.1
67-68	1.432	67-69-71	178.2	66-67-69-71	-174.8
67-69	1.432	67-68-70	178.2	66-67-68-70	-175.5
68-70	1.161	61-60-59	112.6	69-71-68-70	-179.6
69-71	1.160	61-60-59	127.7	63-65-73-74	179.8
73-74	1.335	60-59-58	132.9	64-66-67-68	-2.6
82-83	1.527	57-58-59	132.8	61-60-58-57	-7.6
86-87	1.527	22-21-30	113.1	66-60-58-78	-3.9
90-91	1.527	43-41-50	113.1		
94-95	1.527				

Definition	Bond length	Definition	Bond angles	Definition	Dihedral angles
			IT-2Cl		
1-2	1.741	1-2-3	119.5	8-9-10-11	-2.8
6-7	1.217	7-6-15	120.7	9-10-11-13	-174.6
9-10	1.370	8-9-10	124.4	9-10-12-14	-175.9
10-11	1.432	9-10-11	123.0	11-13-12-14	-179.6
10-12	1.432	9-10-12	124.3	5-3-2-1	179.9
11-13	1.160	10-11-13	178.2	15-9-10-12	-1.1
12-14	1.161	10-12-14	178.2	6-15-17-18	-7.8
15-16	1.370	11-10-12	112.6	9-15-17-19	-4.0
16-17	1.426	6-15-16	127.7	20-21-30-31	-17.4
21-22	1.534	15-16-17	132.8	37-21-30-31	92.3
27-28	1.515	16-17-18	132.7	32-35-36-76	-171.1
28-29	1.527	20-21-22	108.3	35-36-76-84	178.5
29-80	1.528	20-21-30	112.7	36-76-84-85	0
80-81	1.528	37-21-22	113.7	76-84-85-86	-179.2
21-30	1.534	37-21-30	108.5	20-21-22-23	87.8

35-36	1.514	21-22-23	121.5	37-21-22-23	-22.2
36-76	1.527	28-27-26	119.1	26-27-28-29	-172.3
76-84	1.528	21-30-31	121.4	27-28-29-80	178.1
84-85	1.528	36-35-32	119.1	28-29-80-81	179.8
41-43	1.534	42-41-50	112.7	29-80-81-82	-179.5
48-49	1.515	42-41-43	108.3	40-41-50-51	-92.3
49-75	1.527	40-41-50	108.5	42-41-50-51	-17.4
75-88	1.528	40-41-43	113.7	52-55-56-77	-171.1
88-89	1.528	41-50-51	121.4	55-56-77-92	178.5
41-50	1.534	56-55-52	119.1	56-77-92-93	0
55-56	1.514	41-43-44	121.5	77-92-93-94	-179.2
56-77	1.527	49-48-45	119.1	40-41-43-44	-22.2
77-92	1.528	74-73-65	119.5	42-41-43-44	87.8
92-93	1.528	62-61-60	129.4	45-48-49-75	-172.3
58-59	1.426	64-66-67	124.4	48-49-75-88	178.1
59-60	1.370	66-67-69	123.0	49-75-88-89	179.8
61-62	1.217	66-67-68	124.3	75-88-89-90	-179.5
66-67	1.370	69-67-68	112.6	64-66-67-69	175.8
67-68	1.432	67-69-71	178.2	66-67-69-71	-174.6
67-69	1.432	67-68-70	178.2	66-67-68-70	-175.9
68-70	1.161	61-60-59	112.6	69-71-68-70	-179.6
69-71	1.160	61-60-59	127.7	63-65-73-74	179.9
73-74	1.741	60-59-58	132.8	64-66-67-68	-2.8
82-83	1.527	57-58-59	132.7	61-60-58-57	-7.8
86-87	1.527	22-21-30	113.1	66-60-58-78	-4.0
90-91	1.527	43-41-50	113.1		
94-95	1.527				
Definition	Bond length	Definition	Bond angles	Definition	Dihedral angles
			IT-2Br		
1-2	1.888	1-2-3	119.6	8-9-10-11	1.7
6-7	1.217	7-6-15	120.7	9-10-11-13	177.0
9-10	1.370	8-9-10	124.3	9-10-12-14	178.4
10-11	1.432	9-10-11	123.1	11-13-12-14	179.8
10-12	1.432	9-10-12	124.5	5-3-2-1	-179.9
11-13	1.160	10-11-13	178.0	15-9-10-12	0.6
12-14	1.161	10-12-14	178.1	6-15-17-18	3.2
15-16	1.370	11-10-12	112.4	9-15-17-19	1.4
16-17	1.426	6-15-16	127.6	20-21-30-31	-18.8
21-22	1.535	15-16-17	133.2	37-21-30-31	91.2
27-28	1.515	16-17-18	133.1	32-35-36-76	-172.2
28-29	1.527	20-21-22	108.1	35-36-76-84	178.5
29-80	1.528	20-21-30	113.2	36-76-84-85	0
80-81	1.528	37-21-22	113.6	76-84-85-86	-179.2
21-30	1.533	37-21-30	108.6	20-21-22-23	89.2
35-36	1.515	21-22-23	121.8	37-21-22-23	-20.7
36-76	1.527	28-27-26	119.4	26-27-28-29	178.3
76-84	1.528	21-30-31	121.5	27-28-29-80	-179.8
84-85	1.528	36-35-32	119.3	28-29-80-81	179.8
41-43	1.535	42-41-50	113.2	29-80-81-82	179.1
48-49	1.515	42-41-43	108.0	40-41-50-51	91.2
49-75	1.527	40-41-50	108.6	42-41-50-51	-18.8
75-88	1.528	40-41-43	113.6	52-55-56-77	-172.2
88-89	1.528	41-50-51	121.5	55-56-77-92	178.5
41-50	1.533	56-55-52	119.3	56-77-92-93	0
55-56	1.515	41-43-44	121.8	77-92-93-94	-179.2
56-77	1.527	49-48-45	119.4	40-41-43-44	-20.7
77-92	1.528	74-73-65	119.6	42-41-43-44	89.2
92-93	1.528	62-61-60	129.3	45-48-49-75	-178.3
58-59	1.426	64-66-67	124.3	48-49-75-88	-179.8
59-60	1.370	66-67-69	123.1	49-75-88-89	179.8
61-62	1.217	66-67-68	124.5	75-88-89-90	179.7
66-67	1.370	69-67-68	112.4	64-66-67-69	-177.6

67-68	1.432	67-69-71	178.0	66-67-69-71	177.0
67-69	1.432	67-68-70	178.1	66-67-68-70	178.4
68-70	1.161	61-60-59	112.4	69-71-68-70	179.8
69-71	1.160	61-60-59	127.6	63-65-73-74	-179.9
73-74	1.888	60-59-58	133.2	64-66-67-68	1.7
82-83	1.527	57-58-59	133.1	61-60-58-57	3.2
86-87	1.527	22-21-30	112.9	66-60-58-78	1.4
90-91	1.527	43-41-50	112.9		
94-95	1.527				

Definition	Bond length	Definition	Bond angles	Definition	Dihedral angles
			PBDB-T		
2-3	1.463	1-2-3	126.5	1-2-3-4	130.2
42-43	1.501	2-3-4	132.1	3-4-93-94	-7.8
43-44	1.547	4-93-94	121.8	41-42-43-44	131.4
44-45	1.535	42-43-44	116.1	42-43-44-45	-87.9
44-46	1.536	47-48-49	130.1	42-43-44-46	148.8
6-7	1.463	48-49-50	114.5	47-48-49-50	-90.4
10-11	1.454	5-6-7	128.5	48-47-95-96	-17.4
18-19	1.455	6-7-8	130.2	48-49-50-51	55.3
22-23	1.459	9-10-11	130.8	48-49-50-52	178.0
26-27	1.465	10-11-12	125.6	5-6-7-8	-119.4
30-31	1.454	13-14-15	122.1	9-10-11-12	151.1
34-35	1.473	14-15-16	127.9	13-14-15-16	-126.7
38-39	1.473	53-54-55	127.4	53-54-55-56	-103.8
48-49	1.499	54-55-56	116.1	54-55-56-57	-67.1
49-50	1.551	59-60-61	120.5	54-55-56-58	58.6
50-51	1.541	60-61-62	127.2	59-60-61-62	57.5
51-52	1.537	63-64-65	128.0	63-64-65-66	118.8
54-55	1.503	64-65-66	114.8	64-65-66-67	-58.0
55-56	1.546	17-18-19	125.9	64-65-66-68	176.5
56-57	1.535	18-19-20	130.1	17-18-19-20	-141.6
56-58	1.535	21-22-23	128.6	21-22-23-24	140.0
64-65	1.498	22-23-24	130.2	23-24-97-98	-18.2
65-66	1.542	69-70-71	130.4	69-70-71-72	128.9
66-67	1.544	70-71-72	116.1	70-71-72-73	146.8
66-68	1.535	75-76-77	129.7	70-71-72-74	-90.6
70-71	1.502	76-77-78	114.5	75-76-77-78	-90.3
71-72	1.548	25-26-27	128.3	76-77-78-79	177.5
72-73	1.533	26-27-28	130.5	76-77-78-80	54.9
72-74	1.536	29-30-31	131.3	25-26-27-28	-110.6
76-77	1.498	30-31-32	125.4	26-25-99-100	16.8
77-78	1.551	33-34-35	121.8	29-30-31-32	150.0
78-79	1.537	34-35-36	128.3	33-34-35-36	-128.3
78-80	1.541	81-82-83	127.6	81-82-83-84	-104.1
82-83	1.503	82-83-84	116.2	82-83-84-85	60.1
83-84	1.546	37-38-39	122.2	82-83-84-86	-65.3
84-85	1.535	87-88-89	128.2	37-38-39-40	-117.6
84-86	1.535	88-89-90	114.2	87-88-89-90	121.5
88-89	1.500	47-95-96	123.6	88-89-90-91	-56.9
89-90	1.543	24-97-98	122.3	88-89-90-92	177.4
90-91	1.545	25-99-100	122.2		
90-92	1.535				

Table S2. Electronic transition energies (in eV), excitation wavelengths (in nm), excited states characters (ESC), corresponding oscillator strengths (*f*), and the main transition configurations with coefficients larger than 10% for the PBDB-T, ITIC and IT-2X (X= F, Cl, Br). CT (ACT) represents the charge transfer from the donor (acceptor) segment to the acceptor (donor) segment. DLE (ALE) represents the local excitation that occurs on the donor (acceptor) segment. (LC-PBE/6-31G**; α=0.200, β=0.086; ε_s=3.5, ε_d=3.3; ω unit is *Bohr*$^{-1}$).

States	Main transition configurations	ESC	E (eV/nm)	f

PBDB-T (ω = 0.155)				
S1	H→L(83%)	CT&DLE	2.57/482.92	0.9508
S2	H→L+1(53%)	CT	2.75/450.74	0.0225
S3	H-1→L+2(27%);H→L+2(29%); H→L+3(13%)	CT	2.78/446.13	0.1666
S4	H-1→L(73%)	CT&DLE	2.85/434.94	0.0521
S5	H-1→L+2(11%);H→L+1(11%) H→L+2(23%);H→L+3(32%)	CT	2.92/424.74	0.1366
S6	H-1→L+2(35%);H-1→L+3(13%) H→L+2(19%);H→L+3(19%)	CT	3.00/413.85	0.0654
S7	H-2→L(62%)	CT&DLE	3.07/404.48	0.4274
S8	H-3→L+1(10%);H-2→L+1(19%); H→L+1(12%)	CT&ALE	3.13/395.98	0.0429
S9	H-2→L+2(12%);H-1→L+5(24%)	CT&DLE	3.16/392.40	0.0918
S10	H-1→L+1(66%);H→L+1(11%)	CT	3.18/389.65	0.0135
S11	H→L+4(12%)	CT&DLE	3.19/389.08	0.1665
S12	H-3→L(11%);H→L+4(40%)	CT&DLE	3.22/384.49	0.3148
S13	H-1→L+5(30%);H→L+5(13%)	CT&DLE	3.26/380.58	0.2454
S14	H-1→L+3(24%)	CT	3.28/377.52	0.0723
S15	H-1→L+3(35%)	CT	3.30/375.85	0.0379
ITIC (ω = 0.155)				
S1	H→L(96%)	CT&DLE	1.92/646.56	2.8141
S2	H→L+1(96%)	CT&DLE	2.25/551.24	0.0011
S3	H→L+2(91%)	CT&DLE	2.66/466.84	0.1901
S4	H-1→L(93%)	CT&DLE	2.69/460.25	0.0005
S5	H→L+3(87%)	CT&DLE	2.75/451.19	0.0023
S6	H-2→L(91%)	CT&DLE	2.80/443.25	0.0098
S7	H-3→L(86%)	CT&DLE	2.83/437.64	0.0002
S8	H-4→L(78%)	CT&DLE	2.92/424.18	0.3927
S9	H-1→L+1(87%)	CT&DLE	2.95/420.57	0.1265
S10	H-5→L(68%);H-4→L+1(15%)	CT&DLE	2.99/414.24	0.0003
S11	H-7→L+1(10%);H-6→L(67%)	CT&DLE	3.10/400.07	0.0414
S12	H-2→L+1(80%)	CT	3.11/398.07	0.0007
S13	H-3→L+1(87%)	CT	3.17/391.14	0.0010
S14	H-7→L(63%);H-6→L+1(15%)	CT&DLE	3.18/390.11	0.0005
S15	H-15→L(51%);H-14→L+1(25%)	ACT&ALE	3.21/386.07	0.0039
IT-2F (ω = 0.167)				
S1	H→L(96%)	CT&DLE	1.93/644.02	2.8684
S2	H→L+1(96%)	CT&DLE	2.27/546.07	0.0002
S3	H→L+2(90%)	CT&DLE	2.62/472.71	0.1270
S4	H→L+3(82%)	CT&DLE	2.69/461.00	0.0006
S5	H-1→L(86%)	CT&DLE	2.71/457.49	0.0001
S6	H-2→L(91%)	CT&DLE	2.80/443.16	0.0086
S7	H-3→L(86%)	CT&DLE	2.83/437.77	0.0001
S8	H-4→L(80%)	CT&DLE	2.93/423.68	0.3566
S9	H-1→L+1(89%)	CT&DLE	2.97/417.18	0.1644
S10	H-5→L(68%);H-4→L+1(15%)	CT&DLE	3.00/413.03	0.0001
S11	H-7→L+1(10%);H-6→L(66%)	CT&DLE	3.11/399.28	0.0565
S12	H-2→L+1(79%)	CT	3.13/396.61	0.0011
S13	H-3→L+1(87%)	CT	3.18/389.81	0.0015
S14	H-7→L(61%);H-6→L+1(14%)	CT&DLE	3.18/389.57	0.0008
S15	H-16→L+1(12%);H-16→L+3(13%) H-15→L(36%);H-15→L+2(13%)	ACT&ALE	3.26/380.67	0.0003
IT-2Cl (ω = 0.144)				
S1	H→L(96%)	CT&DLE	1.89/657.52	2.8394
S2	H→L+1(96%)	CT&DLE	2.21/559.98	0.0003
S3	H→L+2(91%)	CT&DLE	2.57/483.05	0.1742
S4	H-1→L(28%);H→L+3(64%)	CT&DLE	2.65/468.40	0.0007
S5	H-1→L(67%);H→L+3(26%)	CT&DLE	2.66/465.39	0.0000
S6	H-2→L(90%)	CT&DLE	2.74/452.97	0.0074
S7	H-3→L(86%)	CT&DLE	2.77/447.67	0.0003
S8	H-4→L(82%)	CT&DLE	2.87/432.15	0.3433
S9	H-1→L+1(90%)	CT&DLE	2.91/426.22	0.1671
S10	H-5→L(68%);H-4→L+1(16%)	CT&DLE	2.95/420.01	0.0000
S11	H-7→L+1(11%);H-6→L(67%)	CT&DLE	3.05/406.74	0.0622
S12	H-2→L+1(81%)	CT	3.05/406.00	0.0009
S13	H-3→L+1(87%)	CT	3.10/399.50	0.0023
S14	H-7→L(63%);H-6→L+1(15%)	CT&DLE	3.12/397.71	0.0008

S15	H-5→L(14%);H-4→L+1(54%)	CT&DLE	3.23/384.16	0.0000
IT-2Br (ω = 0.155)				
S1	H→L(96%)	CT&DLE	1.89/657.37	2.8730
S2	H→L+1(96%)	CT&DLE	2.22/559.75	0.0000
S3	H→L+2(91%)	CT&DLE	2.58/480.80	0.1798
S4	H-1→L(60%);H→L+3(34%)	CT&DLE	2.66/466.69	0.0001
S5	H-1→L(35%);H→L+3 (56%)	CT&DLE	2.67/463.89	0.0004
S6	H-2→L(90%)	CT&DLE	2.74/452.97	0.0076
S7	H-3→L(86%)	CT&DLE	2.77/447.13	0.0001
S8	H-4→L(79%)	CT&DLE	2.88/430.87	0.3450
S9	H-1→L+1(90%)	CT&DLE	2.91/425.85	0.1808
S10	H-5→L(67%);H-4→L+1(16%)	CT&DLE	2.95/420.18	0.0012
S11	H-7→L+1(10%);H-6→L(66%)	CT&DLE	3.05/406.04	0.0492
S12	H-2→L+1(82%)	CT	3.05/406.03	0.0005
S13	H-3→L+1(86%)	CT	3.11/399.01	0.0014
S14	H-7→L(65%);H-6→L+1(15%)	CT&DLE	3.12/396.88	0.0004
S15	H-15→L(22%);H-4→L+1(23%)	ACT&ALE	3.23/383.35	0.0011

Table S3. The selected bond lengths (in Å) and bond angles (in °) of PBDB-T:ITIC and PBDB-T:IT-2X (X= F, Cl, Br) complexes. The atomic serial numbers is shown in Figure S1 of supporting information. (ωB97XD/6-31G**).

**Bond length**	**PBDB-T:ITIC**	**PBDB-T:IT-2F**	**PBDB-T:IT-2Cl**	**PBDB-T:IT-2Br**
2-3[a]	1.463	1.462	1.460	1.462
6-7[a]	1.456	1.459	1.458	1.459
10-11[a]	1.451	1.452	1.453	1.451
60-61[a]	1.472	1.473	1.472	1.473
14-15[a]	1.471	1.478	1.473	1.474
18-19[a]	1.457	1.460	1.463	1.457
22-23[a]	1.461	1.458	1.462	1.458
26-27[a]	1.458	1.458	1.455	1.458
30-31[a]	1.452	1.451	1.450	1.451
34-35[a]	1.473	1.473	1.474	1.474
38-39[a]	1.470	1.470	1.471	1.471
1-2[b]	1.086	1.338	1.745	1.887
15-16[b]	1.367	1.367	1.362	1.369
16-17[b]	1.430	1.428	1.432	1.426
58-59[b]	1.430	1.427	1.426	1.428
59-60[b]	1.363	1.367	1.372	1.369
73-74[b]	1.086	1.339	1.742	1.227
Bond angles	PBDB-T:ITIC	PBDB-T:IT-2F	PBDB-T:IT-2Cl	PBDB-T:IT-2Br
1-2-3[a]	126.5	126.8	124.0	126.5
2-3-4[a]	132.3	132.2	134.5	132.4
5-6-7[a]	134.0	134.9	131.9	134.9
6-7-8[a]	123.9	123.8	127.4	124.2
9-10-11[a]	128.6	128.5	130.3	129.6
10-11-12[a]	127.3	127.8	125.8	126.5
59-60-61[a]	121.4	124.5	121.8	122.5
13-14-15[a]	120.8	120.1	120.3	119.4
17-18-19[a]	130.1	127.9	130.0	127.2
18-19-20[a]	124.7	130.0	125.6	128.9
21-22-23[a]	121.0	124.9	122.6	124.2
22-23-24[a]	134.2	134.4	134.5	135.1
25-26-27[a]	135.2	135.1	135.2	135.0
26-27-28[a]	123.5	123.4	122.6	122.6
29-30-31[a]	128.0	128.4	126.5	127.2
30-31-32[a]	127.8	127.3	129.0	128.3
33-34-35[a]	122.1	122.3	122.3	122.4
37-38-39[a]	121.9	122.4	121.6	121.7
1-2-3[b]	120.2	118.7	118.3	119.2
11-10-12[b]	112.1	112.7	113.2	112.3
15-16-17[b]	132.7	131.7	132.4	132.5
74-73-65[b]	120.1	119.0	119.4	119.7
69-67-68[b]	112.7	112.5	112.4	112.7
60-59-58[b]	133.2	133.2	132.4	132.0

[a] represent the atomic serial numbers of the electron donor in the complex.

[b] represent the atomic serial numbers of the electron acceptor in the complex.

Table S4. Electronic transition energies (in eV), corresponding oscillator strengths (f), excitation wavelengths (in nm), excited states characters (ESC) and main transition configurations with coefficients larger than 10% for PBDB-T:ITIC and PBDB-T:IT-2X (X= F, Cl, Br) complexes. (LC-PBE/6-31G**; ε_s=3.5, ε_d=3.3; α=0.200, β=0.086; the ω unit is *Bohr*$^{-1}$).

States	Main transitioncon figurations	ESC	E(eV/nm)	f
	PBDB-T:ITIC (ω = 0.155)			
S1	H→L(75%);H→L+1(16%)	CT	1.60/777.01	0.0522
S2	H-1→L(55%);H-1→L+1(32%)	CT	1.72/719.34	0.1582
S3	H-2→L(75%);H→L+1(13%)	ALE	1.95/636.26	1.9895
S4	H-1→L+1(17%);H→L+1(34%)	CT	2.04/607.81	0.0087
S5	H-4→L(21%);H-4→L+1(10%); H-3→L(33%)	CT	2.10/589.99	0.1993
S6	H-4→L(12%);H-1→L(25%); H-1→L+1(21%)	CT	2.16/574.02	0.0245
S7	H-4→L(26%);H-3→L(14%); H-3→L+1(27%)	CT	2.20/564.08	0.0177
S8	H-2→L+1(16%);H→L+2(45%)	CT&ALE	2.26/548.66	0.0675
S9	H-2→L+1(59%)	ALE	2.30/539.93	0.1396
S10	H-1→L+2(18%);H-1→L+3(25%) H-1→L+4(17%)	CT	2.33/531.14	0.0847
S11	H→L+3(47%);H→L+4(22%)	CT&DLE	2.38/521.46	1.4261
S12	H-5→L(17%);H-3→L(11%); H-3→L+1(35%)	CT&DLE	2.44/508.29	0.0519
S13	H-1→L+4(29%);H-1→L+5(27%); H→L+5(18%)	CT&DLE	2.49/497.93	0.3509
S14	H-2→L+2(21%);H→L+3(12%); H→L+6(17%)	CT&DLE	2.60/477.56	0.0542
S15	H-4→L(14%);H-4→L+1(39%)	CT	2.62/472.56	0.0172
	PBDB-T:IT-2F (ω = 0.167)			
S1	H-1→L(21%);H-1→L+1(14%); H→L(60%)	CT	1.71/723.8	0.0398
S2	H-1→L(42%);H→L(14%); H→L+1(37%)	CT	1.79/694.36	0.1136
S3	H-2→L(88%)	ALE	1.91/647.97	2.1042
S4	H-3→L(29%);H-1→L+1(30%)	CT	2.11/588.93	0.0823
S5	H-4→L(32%);H-3→L(13%); H-2→L+1(12%)	CT	2.15/576.07	0.0103
S6	H-1→L(19%);H→L(12%); H→L+1(32%)	CT	2.20/564.47	0.0236
S7	H-4→L(12%);H-2→L+1(61%)	ALE	2.25/552.09	0.0213
S8	H-4→L(19%);H-3→L+1(11%) H-1→L+2(25%);H→L+2(22%)	CT&DLE	2.28/544.19	0.3681
S9	H-3→L(21%);H-1→L+1(19%) H-1→L+2(13%);H→L+2(11%)	CT	2.31/535.86	0.1641
S10	H-1→L+1(11%);H-1→L+3(18%); H→L+3(35%)	CT	2.33/531.55	0.0006
S11	H-1→L+5(12%);H→L+3(10%); H→L+4(46%)	DLE	2.43/509.86	1.5504
S12	H-1→L+5(16%);H→L+4(14%); H→L+5(35%)	DLE	2.47/501.71	0.1341
S13	H-3→L(12%);H-3→L+1(30%); H-2→L+2(23%)	CT&ALE	2.51/493.35	0.0295
S14	H-3→L+1(14%);H-2→L+2(54%)	ALE	2.55/487.04	0.0894
S15	H-2→L+3(60%)	ALE	2.58/479.82	0.0048
	PBDB-T:IT-2Cl (ω = 0.144)			
S1	H→L(79%);H→L+1(16%)	CT	1.47/844.09	0.0212
S2	H-1→L(59%);H-1→L+1(27%)	CT	1.62/767.22	0.0471
S3	H-2→L(89%)	ALE	1.89/654.39	2.1403
S4	H-3→L(64%);H-1→L+1(11%)	CT	1.90/651.96	0.0359
S5	H-1→L(14%);H→L+1(60%)	CT	1.99/623.77	0.0037
S6	H-3→L(15%);H-1→L(16%); H-1→L+1(41%)	CT	2.08/594.93	0.0094
S7	H→L+2(61%);H→L+3(12%)	CT	2.13/580.78	0.0796

S8	H-4→L(32%);H-4→L+1(16%); H-2→L+1(28%)	CT	2.21/560.81	0.0293
S9	H-2→L+1(21%);H-1→L+2(13%); H-1→L+3(44%)	CT&DLE	2.23/555.36	0.0503
S10	H-4→L(20%);H-2→L+1(42%)	CT&ALE	2.27/547.09	0.0586
S11	H-3→L+1(59%)	CT	2.37/522.75	0.0176
S12	H→L+4(23%);H→L+5(62%)	DLE	2.42/513.06	1.2822
S13	H-1→L+4(57%);H→L+5(15%)	DLE	2.50/494.97	0.4672
S14	H-3→L+2(22%);H-2→L+2(27%); H-1→L+2(23%)	CT&ALE	2.53/489.56	0.0136
S15	H-5→L(16%);H-2→L+2(13%); H→L+3(26%)	CT	2.58/481.29	0.0068
PBDB-T:IT-2Br (ω = 0.155)				
S1	H→L(75%)	CT	1.62/767.38	0.0266
S2	H-1→L(48%);H-1→L+1(14%); H→L+1(28%)	CT	1.74/712.86	0.0959
S3	H-2→L(90%)	ALE	1.87/662.95	2.0919
S4	H-4→L(19%);H-1→L+1(20%); H→L+1(26%)	CT	2.03/609.98	0.1893
S5	H-4→L(11%);H-4→L+1(13%) H-3→L(50%);H-2→L+1(14%)	CT	2.07/599.06	0.0002
S6	H-4→L(15%);H-1→L(24%); H→L+1(31%)	CT	2.14/579.48	0.0029
S7	H-2→L+1(45%);H-1→L+1(22%)	CT&ALE	2.18/569.98	0.0153
S8	H-4→L(11%);H-1→L+2(15%); H→L+2(51%)	CT&DLE	2.21/561.55	0.4839
S9	H-3→L(20%);H-3→L+1(11%) H-2→L+1(20%);H-1→L+1(13%)	CT&DLE	2.24/552.67	0.0359
S10	H-1→L+3(37%);H→L+3(27%)	CT	2.30/540.04	0.0021
S11	H→L+3(11%);H→L+4(62%)	DLE	2.39/518.12	1.4911
S12	H-4→L+1(14%);H-3→L+1(27%)	CT	2.46/503.81	0.0258
S13	H-2→L+2(13%);H-1→L+5(16%); H→L+5(33%)	DLE	2.50/496.5	0.4795
S14	H-2→L+2(52%);H-2→L+3(11%)	ALE	2.51/493.5	0.0187
S15	H-2→L+2(11%);H-2→L+3(56%)	ALE	2.53/489.5	0.0198

Table S5. Table 2. X (X= F, Cl, Br) molecules. ($U_1 = \sqrt{X^2+Y^2+Z^2}$; LC-PBE/6-31G**; ω=0.155, 0.167, 0.144, 0.155 *Bohr*$^{-1}$; α=0.200, β=0.086; ε_s=3.5,ε_d=3.3)

	Dipole moment			
	X	Y	Z	U_1
**ITIC**	0.0001	0.0003	-1.9992	1.9992
IT-2F	-0.0009	0.0001	2.8949	2.8949
IT-2Cl	-0.0002	0.0000	2.8763	2.8763
IT-2Br	-0.0001	0.0000	-1.5954	1.5954

	Quadrupole moment					
	XX	YY	ZZ	XY	XZ	YZ
ITIC	-777.4210	-562.5458	-595.9622	-98.3005	-0.0053	0.0014
IT-2F	-801.0509	-612.3295	-605.6509	-65.3123	-0.0157	-0.0031
IT-2Cl	-826.4463	-643.5688	-622.8386	-57.6721	-0.0025	-0.0006
IT-2Br	-818.2559	-665.1685	-631.8976	-83.6713	0.0058	-0.0001

Table S6. Calculated electronic coupling V (in eV) in CR process of PBDB-T:ITIC and PBDB-T:IT-2X (X= F, Cl, Br) complexes. u correspond the transition dipole moment (in a.u); μ_g and μ_e represent the dipole moment at the ground state and excited state (in Debye), respectively. $\Delta\mu_{ge}$ indicates the change in dipole moment between the ground state and the lowest singlet excited state (in Debye); ΔE is the energy differnence between the ground state and the excited state (in eV). (LC-PBE/6-31G**; ω=0.155, 0.167, 0.144, 0.155 *Bohr*$^{-1}$; α=0.200, β=0.086; ε_s=3.5, ε_d=3.3)

		**PBDB-T :ITIC**	**PBDB-T :IT-2F**	**PBDB-T :IT-2Cl**	**PBDB-T :IT-2Br**
u	X	1.1247	-0.9862	-0.7703	0.7873

	Y	0.1391	-0.0360	-0.0599	0.1274
	Z	0.2874	0.0395	0.0830	0.2204
U_g	X_1	0.7576	-0.3990	-0.0380	-0.9073
	Y_1	0.3824	-0.6535	0.1921	0.0771
	Z_1	6.5663	1.5759	2.1197	-1.3897
U_e	X_2	-0.2984	0.2780	5.5942	-0.5185
	Y_2	2.4181	1.5862	10.4223	-0.4372
	Z_2	-4.5896	-10.2784	-8.4810	-13.6932
u		1.1691	0.9876	0.7771	0.8274
$\Delta\mu_{ge}$		11.3892	12.0830	15.7719	12.3149
ΔE		1.5957	1.7130	1.4689	1.6157
V_{CR}		0.3691	0.3287	0.1785	0.2611

$$\Delta\mu_{ge} = \sqrt{(X_2 - X_{1)}{}^2 + (Y_2 - Y_{1)}{}^2 + (Z_2 - Z_{1)}{}^2} \qquad u = \sqrt{X^2 + Y^2 + Z^2}$$

Table S7. Electronic coupling V (in meV) in CT and ED process of PBDB-T:ITIC and PBDB-T:IT-2X (X= F, Cl, Br) complexes. E_i and E_f are the energy (in eV) of initial and final states; S_{if} covresponds the overlap matrix element, and H_{if} (meV) is the off-diagonal hamiltonian of charge-localized state. (LC-PBE/6-31G**; ω=0.155, 0.167, 0.144, 0.155 *Bohr*$^{-1}$; α=0.200, β=0.086; ε_s=3.5, ε_d=3.3).

Complex	ETP	H_{if}	S_{if}	E_i	E_f	V
PBDB-T:ITIC	CT D(H)→A(L)	253.780	-0.018	-5.354	-3.174	178.951
	ED A(H)→D(H)	67.841	-0.006	-5.357	-5.641	37.562
PBDB-T:IT-2F	CT D(H)→A(L)	216.863	-0.015	-5.396	-3.155	153.234
	ED A(H)→D(H)	26.529	-0.002	-5.398	-5.622	18.190
PBDB-T:IT-2Cl	CT D(H)→A(L)	70.97704	-0.004	-5.38302	-3.26686	52.70569
	ED A(H)→D(H)	99.768	-0.007	-5.383	-5.692	59.840
PBDB-T:IT-2Br	CT D(H)→A(L)	-67.943	0.005	-5.377	-3.217	47.065
	ED A(H)→D(H)	-42.055	0.003	-5.377	-5.636	26.231
